# Presynaptic Gq-coupled receptors drive biphasic dopamine transporter trafficking that modulates dopamine clearance and motor function

**DOI:** 10.1016/j.jbc.2023.102900

**Published:** 2023-01-12

**Authors:** Patrick J. Kearney, Nicholas C. Bolden, Elizabeth Kahuno, Tucker L. Conklin, Gilles E. Martin, Gert Lubec, Haley E. Melikian

**Affiliations:** 1 Brudnick Neuropsychiatric Research Institute, Department of Neurobiology, UMASS Chan Medical School, Worcester, Massachusetts, USA; 2 Morningside Graduate School of Biomedical Sciences, UMASS Chan Medical School, Worcester, Massachusetts, USA; 3 Department of Neuroproteomics, Paracelsus Private Medical University, Salzburg, Austria

**Keywords:** dopamine, metabotropic glutamate receptor, membrane trafficking, striatum, motor function, AAV, adeno-associated virus, ACSF, artificial cerebrospinal fluid, CNO, clozapine-*N*-oxide, DA, dopamine, DAT, DA transporter, DHPG, (*RS*)-3,5-dihydroxyphenylglycine, DRD2, D2 DA receptor, DRD2auto, DRD2 autoreceptor, DS, dorsal striatal, eGFP, enhanced GFP, FSCV, fast-scan cyclic voltammetry, GPCR, G protein–coupled receptor, MTEP, 3-[(2-methyl-1,3-thiazol-4-yl)ethynyl]-pyridine, PD, Parkinson’s disease, qPCR, quantitative PCR, TH, tyrosine hydroxylase, VS, ventral striata, VTA, ventral tegmental area

## Abstract

Extracellular dopamine (DA) levels are constrained by the presynaptic DA transporter (DAT), a major psychostimulant target. Despite its necessity for DA neurotransmission, DAT regulation *in situ* is poorly understood, and it is unknown whether regulated DAT trafficking impacts dopaminergic signaling and/or behaviors. Leveraging chemogenetics and conditional gene silencing, we found that activating presynaptic Gq-coupled receptors, either hM3Dq or mGlu5, drove rapid biphasic DAT membrane trafficking in *ex vivo* striatal slices, with region-specific differences between ventral and dorsal striata. DAT insertion required D2 DA autoreceptors and intact retromer, whereas DAT retrieval required PKC activation and Rit2. *Ex vivo* voltammetric studies revealed that DAT trafficking impacts DA clearance. Furthermore, dopaminergic mGlu5 silencing elevated DAT surface expression and abolished motor learning, which was rescued by inhibiting DAT with a subthreshold CE-158 dose. We discovered that presynaptic DAT trafficking is complex, multimodal, and region specific, and for the first time, we identified cell autonomous mechanisms that govern presynaptic DAT tone. Importantly, the findings are consistent with a role for regulated DAT trafficking in DA clearance and motor function.

Dopamine (DA) is critical for movement, learning, motivation, and reward (1, 2), and DAergic dysfunction is implicated in multiple neuropsychiatric disorders including Parkinson’s disease (PD), attention-deficit hyperactivity disorder, schizophrenia, and addiction (3, 4, 5, 6). Following its release, DA is temporally and spatially constrained by the presynaptic DA transporter (DAT), which recaptures extracellular DA (7). The central role of DAT in DAergic transmission is illustrated by the consequences of pharmacologically or genetically silencing DAT (8). For example, DAT is the primary target for addictive and therapeutic psychostimulants, such as cocaine, methylphenidate (Ritalin), and amphetamine. These agents markedly enhance extracellular DA through their actions as DAT inhibitors (cocaine and methylphenidate) and substrates (amphetamine). Moreover, multiple DAT coding variants have been reported in probands from attention-deficit hyperactivity disorder, and autism spectrum disorder patients (9, 10, 11, 12, 13, 14), as well as in DAT deficiency syndrome, a form of Parkinsonism (15, 16, 17, 18, 19). Given that DAT function profoundly impacts DAergic signaling, it is vital that we understand the molecular mechanisms that acutely regulate DAT availability. Such information may inform potential interventions for DA-related neuropsychiatric disorders.

Extensive research from multiple laboratories supports that DAT surface expression is regulated by membrane trafficking (20, 21, 22, 23). DAT constitutively cycles between the plasma membrane and endosomes. Direct PKC activation accelerates DAT endocytosis, and thereby diminishes DAT surface levels and function (24, 25, 26). PKC-stimulated DAT internalization requires the neuronal GTPase Rit2 (27), whereas DAT endocytic recycling requires the Vps35^+^ retromer complex (28, 29). In previous studies, PKC was directly activated with phorbol esters or *via* a Gq-coupled receptor in non-DAergic cell lines. However, it is still unknown whether, or how, DAT traffics in *bona fide* DAergic terminals in response to endogenous receptor-mediated PKC activation. Furthermore, it is unknown whether regulated DAT trafficking is regionally distinct or whether DAT trafficking impacts DAergic signaling and DA-dependent behaviors. In this study, we leveraged chemogenetics and *in vivo* conditional gene silencing, complemented by pharmacological, electrochemical, and behavioral approaches, to directly probe these questions. Our results demonstrate that regulated DAT trafficking is significantly more complex than had been previously appreciated and that mechanisms that perturb DAT trafficking also dysregulate DA signaling and DA-dependent motor behaviors.

## Results

### Gq-coupled DREADD activation drives region-specific and biphasic DAT trafficking

**Figure 1:**
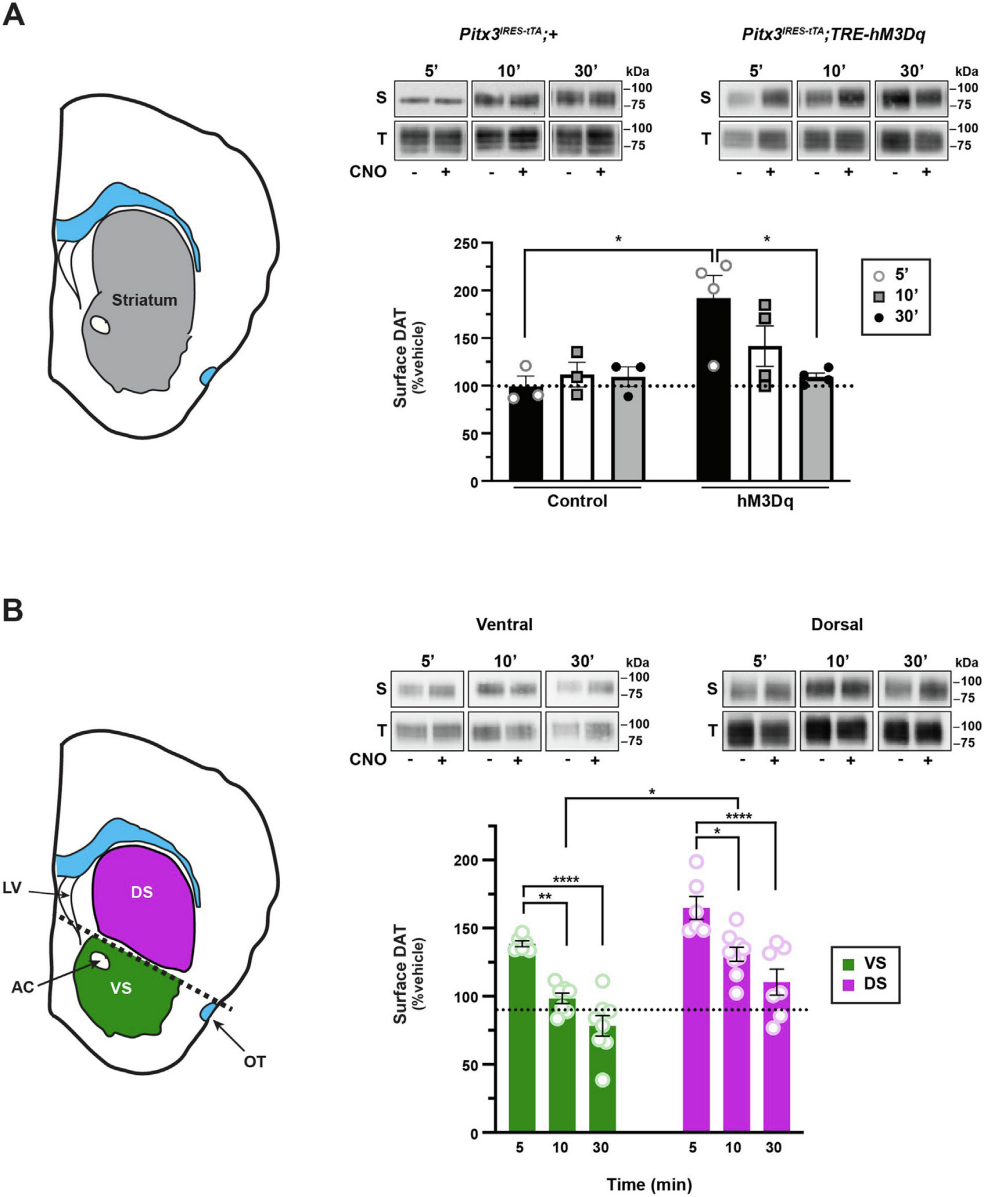
**Gq-coupled DREADD activation drives region-specific and biphasic DAT trafficking**. *Ex vivo* striatal slice surface biotinylation. Acute striatal slices prepared from *Pitx3*^*IRES-tTA*^*;+* or *Pitx3*^*IRES-tTA*^*;TRE-hM3Dq* mice were treated ±500 nM CNO for 5, 10, or 30 min, and DAT surface levels were measured by surface biotinylation as described in Experimental procedures. *A*, *total striatum.* Total striatal slices (*left*) were assessed for hM3Dq-mediated DAT trafficking. *Right, top*, representative immunoblots showing surface (S) and total (T) DAT bands. *Right, bottom*, mean DAT surface levels are presented as %vehicle-treated contralateral hemisection ±SEM. Two-way ANOVA: interaction: *F*_(2,15)_ = 3.82, ∗*p* = 0.045; genotype: *F*_(1,15)_ = 8.53, ∗*p* = 0.011, time: *F*_(2,15)_ = 2.24, *p* = 0.24. ∗*p* < 0.05, Tukey’s multiple comparisons test, n = 3 (*Pitx3*^*IRES-tTA*^) and 4 (*Pitx3*^*IRES-tTA*^*;TRE-hM3Dq*). *B*, *subdissected striatum. Left*, dorsal and ventral striata were subdissected prior to solubilizing, as described in Experimental procedures. *Right*, *top*, representative immunoblots showing surface (S) and total (T) DAT bands. *Right bottom*, mean DAT surface levels, presented as %vehicle-treated contralateral hemisection ±SEM. Two-way ANOVA: interaction: (*F*_(2,37)_ = 0.12, *p* = 0.89; time: *F*_(2,37)_ = 34.65, ∗∗∗∗*p* < 0.0001, region: *F*_(1,37)_ = 30.05, ∗∗∗∗*p* < 0.0001). ∗∗*p* = 0.003; ∗*p* = 0.011. Tukey’s multiple comparisons test. *Ventral*: n = 6 (5 min), 7 (10 min), and 8 (30 min); *dorsal:* n = 6 (5 min), 9 (10 min), and 8 (30 min). CNO, clozapine-*N*-oxide; DAT, dopamine transporter.

We first aimed to test whether cell-autonomous and presynaptic Gq-coupled signaling impacts DAT surface availability in *bona fide* striatal DAergic terminals. To selectively activate Gq-coupled receptors on DAergic terminals, we leveraged the Tet-OFF system to conditionally express the Gq-coupled DREADD, hM3Dq, in DA neurons of *Pitx3*^*IRES-tTA*^*;TRE-HA-hM3Dq* mice (30). Pitx3 is a DA-specific transcription factor, and previous work from our laboratory and others demonstrated that *Pitx3*^*IRES-tTA*^ selectively drives gene expression in midbrain DA neurons (31, 32). We used surface biotinylation to ask whether DAergic hM3Dq activation modulates DAT surface expression in *ex vivo* striatal slices, containing both ventral striata (VS) and dorsal striata (DS). Treatment with the DREADD-specific agonist, CNO (30), (500 nM, 37 °C) biphasically modulated DAT surface expression, which significantly increased by 5 min, and returned to baseline by 30 min (Fig. 1*A*). Importantly, CNO had no significant effect on DAT surface levels in striatal slices from control *Pitx3*^*IRES-tTA*^*/+* littermates (Fig. 1*A*). We further asked whether Gq-stimulated DAT trafficking was either sex- or region-specific in VS and DS subregions. hM3Dq-stimulated DAT trafficking did not significantly differ between males and females (two-way ANOVA, no effect of sex, *p* = 0.59 [DS]; *p* = 0.36 [VS], n = 8 [males], n = 6 [females]); therefore, data from male and female mice were pooled. Similar to total striatum, DAT surface expression rapidly increased in both VS and DS plasma membranes in response to CNO treatment (500 nM, 5 min, 37 °C, Fig. 1*B*). In VS, DAT surface expression significantly diminished to baseline levels by 10 min (Fig. 1*B*). In contrast, in DS, surface DAT remained elevated after 10 min but returned to baseline by 30 min. DAT surface expression in DS remained significantly higher than VS at both 10 and 30 min (Fig. 1*B*).

### Biphasic Gq-stimulated DAT trafficking is mediated by D2 DA receptor and PKC

Gq-stimulated and biphasic DAT trafficking was surprising in light of copious previous reports from both our laboratory (24, 25, 27, 29, 33) and others (21, 34) that direct PKC activation stimulates DAT internalization and decreases DAT surface expression. Moreover, Gq-coupled receptor activation in non-DAergic cells and midbrain decreases DAT surface levels in a PKC-dependent manner (34, 35). Since our current studies tested Gq-stimulated DAT trafficking in DAergic terminals, we considered what factors, specific to DAergic terminals, might mediate biphasic DAT trafficking. Previous studies independently reported that hM3Dq activation evokes DA release (36, 37, 38), and that the D2 DA receptor (DRD2) drives DAT insertion in synaptosomes (39, 40, 41). Therefore, we hypothesized that (1) initial DAT membrane insertion may be due to hM3Dq-stimulated DA release and subsequent DRD2 autoreceptor (DRD2_auto_) activation and (2) DAT return to baseline is mediated by PKC-dependent DAT internalization, acting as a retrieval mechanism following enhanced DAT surface delivery.

**Figure 2:**
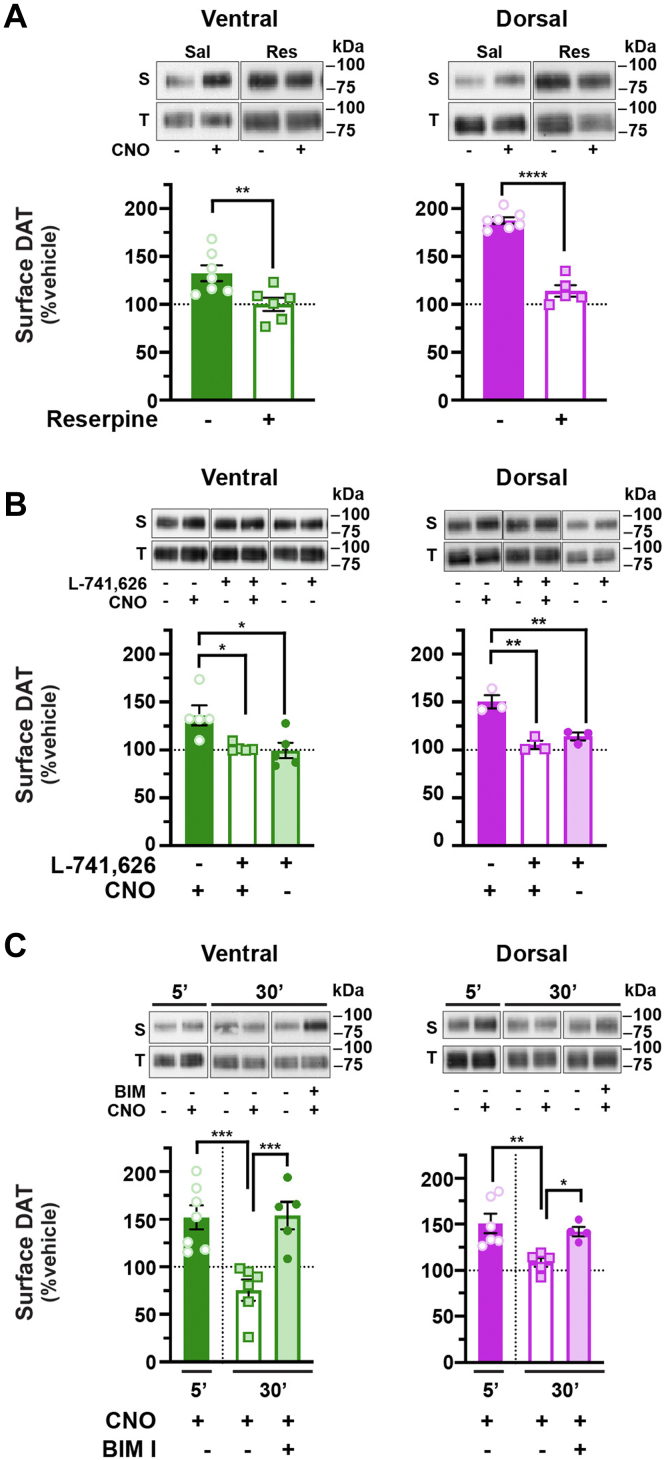
**DA release and DRD2 activation are required for Gq-stimulated DAT insertion, whereas PKC activity is required for DAT retrieval**. *Ex vivo* striatal slice surface biotinylation. Acute striatal slices prepared from *Pitx3*^*IRES-tTA*^*;TRE-hM3Dq* mice were treated with the indicated drugs for the indicated times. DAT surface levels were measured by slice biotinylation, and VS and DS were isolated prior to tissue lysis as described in Experimental procedures. Mean DAT surface levels in ventral striata (VS; *left*) and dorsal striata (DS; *right*) are presented as %vehicle-treated ±SEM, determined as described in Experimental procedures. Representative blots containing both surface (S) and total (T) DAT are shown above each graph, for all treatments and were taken from a single immunoblot exposure, cropped for presentation purposes, with molecular weight markers indicating kilodaltons (kDa). *A*, *reserpine treatment.* Mice were injected (I.P.) with either saline (Sal) or 5.0 mg/kg reserpine (Res) 16 h prior to preparing slices, and slices were treated ±1.0 μM reserpine throughout the experiment. Slices were treated ±500 nM CNO, 5 min, 37 °C. *Ventral:* ∗∗*p* = 0.007, one-tailed, unpaired Student’s *t* test, n = 8 (saline) and 6 (reserpine). *Dorsal:* ∗∗∗∗*p* < 0.0001, one-tailed, unpaired Student’s *t* test, n = 7 (saline) and 5 (reserpine). *B*, *DRD2 antagonist pretreatment.* Striatal slices were pretreated ±DRD2 antagonist (L-741,626, 25 nM, 15 min, 37 °C) and then treated ±500 nM CNO (5 min, 37 °C). DRD2 blockade abolished hM3Dq-stimulated DAT membrane insertion but had no effect alone in either VS (*left*) or DS (*right*). *Ventral:* Kruskal–Wallis test, 8.82. ∗*p* < 0.05, Dunn’s multiple comparisons test, n = 5. *Dorsal:* one-way ANOVA, *F*_(2,6)_ = 20.17, ∗∗*p* = 0.002. ∗∗*p* < 0.01, Bonferroni’s multiple comparisons test, n = 3. *C*, *PKC inhibition.* Slices were pretreated ±500 nM CNO (5 min, 37 °C), followed by treatment ±1.0 μM BIM I (25 min, 37 °C), and DAT surface levels were measured by slice biotinylation at either 5 or 30 min post-CNO treatment. BIM I significantly blocked DAT retrieval following CNO-stimulated membrane insertion in both VS and DS. *Ventral:* one-way ANOVA: *F*_(3,19)_ = 9.33, ∗∗∗*p* = 0.0005. ∗∗∗*p* < 0.002, Bonferroni’s multiple comparisons test, n = 5 to 7. *Dorsal:* one-way ANOVA: *F*_(3,14)_ = 9.41, ∗∗*p* = 0.001. ∗*p* = 0.04, ∗∗*p* = 0.004, Bonferroni’s multiple comparisons test, n = 6 to 7. CNO, clozapine-*N*-oxide; DA, dopamine; DAT, dopamine transporter; DRD2, D2 DA receptor.

To test these hypotheses, we first asked whether DA release is required for hM3Dq-stimulated DAT insertion. To block DA release, we depleted vesicular DA stores with a single reserpine injection (5 mg/kg, I.P.) 16 h prior to preparing striatal slices, which is sufficient to block evoked DA release (42, 43). Vesicular DA depletion completely abolished hM3Dq-stimulated DAT insertion in response to a 5 min CNO treatment, in both VS and DS, as compared with slices from saline-injected mice (Fig. 2*A*). Interestingly, reserpine treatment also increased basal DAT surface expression in the DS but not VS (Fig. S1*A*). These results demonstrate that vesicular DA release is required for rapid hM3Dq-stimulated increase in DAT surface expression.

Given that DAT surface delivery following hM3Dq activation required DA release, we next asked whether DRD2 activation is required for hM3Dq-stimulated DAT insertion in VS and DS and/or is itself sufficient to drive rapid DAT insertion. hM3Dq-expressing striatal slices were pretreated with the DRD2-specific antagonist L-741,626 (44) (25 nM, 15 min, 37 °C), and hM3Dq-stimulated DAT insertion was evoked with CNO (500 nM, 5 min, 37 °C). DRD2 inhibition completely abolished CNO-stimulated DAT insertion in both VS and DS as compared with either vehicle-treated slices or slices treated with L-741,626 alone (Fig. 2*B*). Furthermore, in wildtype mouse slices treated with the DRD2 selective agonist sumanirole (45) (170 nM, 5 min, 37 °C), DAT surface levels significantly increased in both VS and DS (Fig. S1*B*). Enhanced DAT surface levels were sustained in the DS out to 30 min but rapidly returned to baseline by 10 min in the VS (Fig. S1*B*). Previous studies reported that DRD2-stimulated DAT insertion in striatal synaptosomes requires PKCβ activity (39). To test whether hM3Dq-stimulated DAT insertion was similarly dependent upon PKCβ activity, we pretreated slices with the PKCβ-specific inhibitor ruboxistaurin (46, 47) (50 nM, 30 min, 37 °C), prior to activating hM3Dq with CNO. Ruboxistaurin pretreatment completely abolished CNO-stimulated DAT insertion as compared with vehicle-pretreated slices (Fig. S1*C*), consistent with DRD2-mediated and PKCβ-dependent DAT membrane delivery. Taken together, these data demonstrate that both DA release and DRD2 activation are required for hM3Dq-stimulated DAT membrane insertion in VS and DS.

After interrogating the mechanisms of hM3Dq-mediated DAT insertion, we next investigated whether subsequent DAT return to baseline requires PKC. To test this, we first induced DAT insertion with CNO (500 nM, 5 min) and then treated slices with ±1 μM BIM I, a PKC-specific inhibitor. Although CNO treatment alone drove biphasic DAT insertion and subsequent retrieval in control slices, BIM I treatment after DAT insertion significantly abolished DAT return to baseline in both VS and DS (Fig. 2*C*), consistent with the premise that DAT retrieval following hM3Dq-stimulated insertion requires PKC activity. BIM I treatment alone had no effect on DAT surface expression (Fig. S1*D*), suggesting that there is little tonic PKC activity in *ex vivo* DAergic terminals. Taken together, these results clearly demonstrate that presynaptic Gq-coupled receptor activation drives biphasic DAT trafficking that is facilitated by Gq-stimulated DA release, DRD2-mediated DAT insertion, and PKC-dependent DAT membrane retrieval.

### Presynaptic mGlu5 drives DAT trafficking and influences DAT surface tone

**Figure 3:**
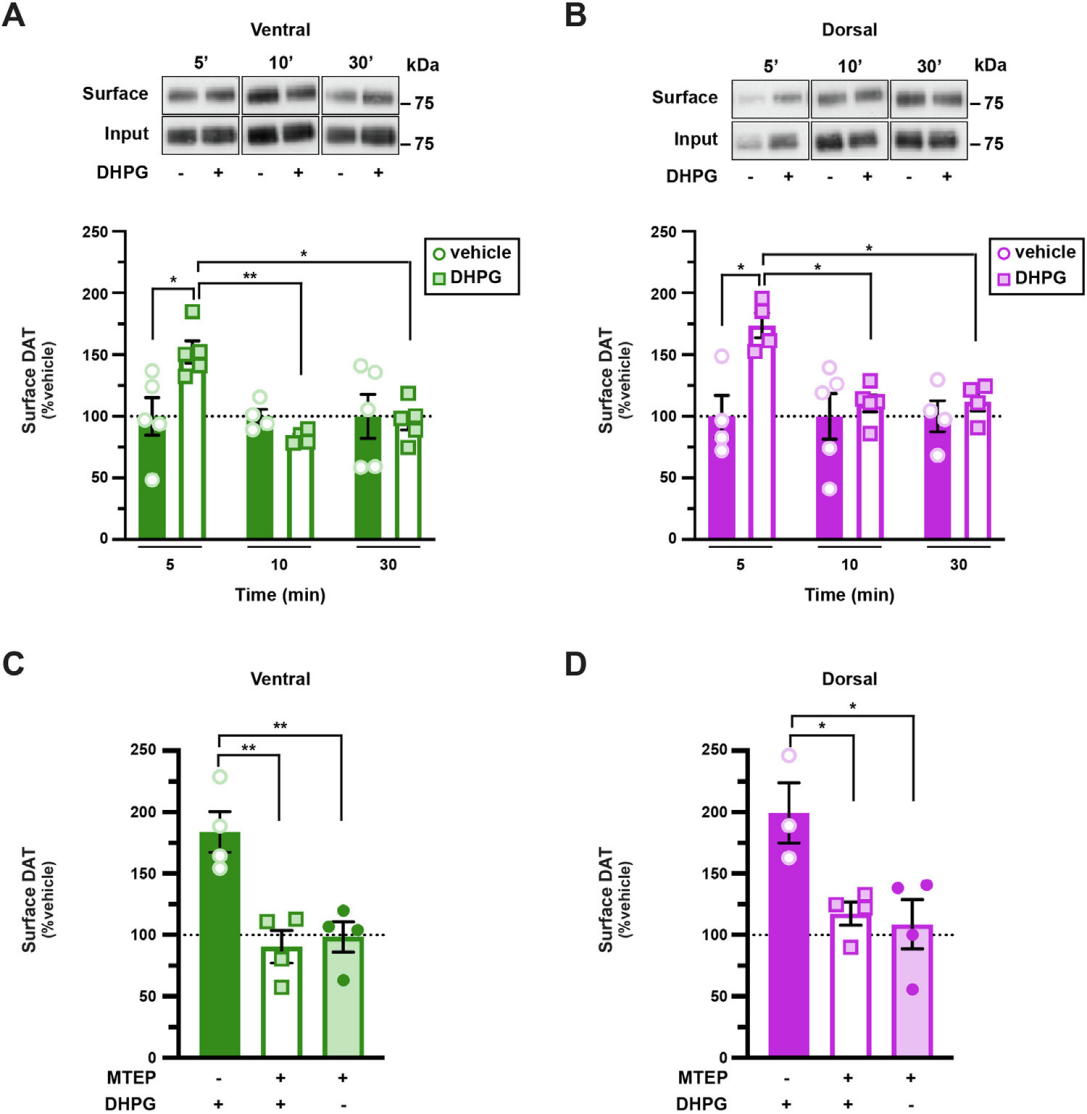
**Striatal mGlu5 activation drives biphasic DAT trafficking**. *Ex vivo striatal slice surface biotinylation.* Acute striatal slices prepared from male C57Bl/6J mice were treated with the indicated drugs for the indicated times. DAT surface levels were measured by slice biotinylation, dorsal and ventral striata were subdissected, and surface DAT was quantified as described in Experimental procedures. Representative blots containing both surface (S) and total (T) DAT are shown above each graph, for all treatments. Average surface DAT values are expressed as % vehicle-treated contralateral hemisection ±SEM. *A* and *B*, *DHPG treatment.* Slices were treated ±10 μM DHPG for 5, 10, or 30 min. *A*, *ventral:* two-way ANOVA: interaction: *F*_(2,22)_ = 4.95, ∗*p* = 0.02; time: *F*_(2,22)_ = 4.95, ∗*p* = 0.02, drug: *F*_(1,22)_ = 2.24, *p* = 0.29. ∗*p* < 0.05, ∗∗*p* = 0.006, Tukey’s multiple comparisons test, n = 4 to 5. *B, dorsal:* two-way ANOVA: interaction: *F*_(2,20)_ = 3.65, ∗*p* = 0.04; time: *F*_(2,20)_ = 3.65, ∗*p* = 0.04, drug: *F*_(1,20)_ = 8.80, ∗∗*p* = 0.008. ∗*p* < 0.05, Tukey’s multiple comparisons test, n = 4 to 5. *C* and *D*, *mGlu5 antagonist pretreatment.* Slices were pretreated ±MTEP (50 nM, 15 min, 37 °C), a selective mGlu5 antagonist, prior to stimulating DAT insertion with DHPG (10 μM, 5 min, 37 °C). MTEP pretreatment abolished DHPG-stimulated DAT membrane insertion in both ventral (*C*) and dorsal (*D*) striata. *Ventral:* one-way ANOVA: *F*_(2,9)_ = 13.42, ∗∗*p* = 0.002; ∗∗*p* < 0.01, Bonferroni’s multiple comparisons test, n = 4. *Dorsal:* one-way ANOVA: *F*_(2,8)_ = 6.92, ∗*p* = 0.02. ∗*p* < 0.05, Bonferroni’s multiple comparisons test, n = 3 to 4. DAT, dopamine transporter; DHPG, (RS)-3,5-dihydroxyphenylglycine; MTEP, 3-[(2-methyl-1,3-thiazol-4-yl)ethynyl]-pyridine.

We next asked whether a native, presynaptic, Gq-coupled receptor similarly drives biphasic DAT trafficking. Group I metabotropic glutamate receptors (mGlu1 and mGlu5) are Gq-coupled G protein–coupled receptors (GPCRs) that are highly expressed in striatum and midbrain DA neurons (48, 49, 50). Moreover, a previous report found that mGlu5 activation functionally downregulated DAT in striatal synaptosomes (51). To test whether group I mGluRs regulate DAT surface expression, we treated *ex vivo* striatal slices from wildtype mice with group I mGluR agonist, (*RS*)-3,5-dihydroxyphenylglycine (DHPG) (10 μM, 37 °C). Similar to our results with hM3Dq, DHPG treatment significantly increased DAT surface levels at 5 min in both VS and DS (Fig. 3, *A* and *B*). However, we did not observe region-specific DAT retrieval kinetics following DHPG treatment, and DAT was significantly retrieved from the membrane by 10 min in both VS and DS (Fig. 3, *A* and *B*). DHPG-mediated effects on DAT surface expression were not because of an overall increase in total DAT (Fig. S2*A*) nor were they because of generalized membrane trafficking effects, as DHPG treatment for 5 min, 37 °C did not significantly affect transferrin receptor surface expression (Fig. S2*B*). To test whether DHPG-stimulated DAT insertion at 5 min was specifically mediated by mGlu5, mGlu1, or both, we pretreated slices with the mGlu5-specific antagonist 3-[(2-methyl-1,3-thiazol-4-yl)ethynyl]-pyridine (52) (50 nM, 15 min) prior to DHPG treatment. 3-[(2-Methyl-1,3-thiazol-4-yl)ethynyl]-pyridine (MTEP) pretreatment completely abolished DHPG-stimulated DAT surface increases at 5 min in both VS and DS, as compared with slices treated with DHPG alone (Fig. 3, *C* and *D*), consistent with an mGlu5-, but not mGlu1-, mediated mechanism.

mGlu5 is widely expressed in the striatum and reportedly expressed in midbrain neurons. However, it is unknown whether mGlu5 is expressed in DAergic terminals; thus, it is unclear whether DHPG stimulates DAT trafficking directly *via* a presynaptic mGlu5 or indirectly *via* mGlu5s expressed elsewhere within the striatum. We previously leveraged the Tet-OFF system as a means to achieve adeno-associated virus (AAV)–mediated conditional gene silencing in DA neurons (29, 32) and aimed to use this approach to conditionally silence mGlu5 in DA neurons. We previously demonstrated that AAV9 injection into *Pitx3*^*IRES-tTA*^ mouse ventral tegmental area (VTA) transduced DA neurons in both VTA and substantia nigra pars compacta and achieved robust gene silencing in substantia nigra pars compacta but not in the neighboring non-DAergic SN reticulata, confirming both the viral spread and selectivity for DA neurons (29, 32). To further assure that injecting our AAV9 vectors into VTA efficaciously transduced nigrostriatal projections into the DS, as well as mesolimbic projections to the VS, we assessed striatal GFP reporter expression encoded in our AAV9 vectors. AAV9 particle injection into VTA resulted in widespread and robust GFP expression throughout both DS and VS, which colocalized with tyrosine hydroxylase (TH+) terminals, but was not apparent in adjacent areas (Fig. S2*C*).

**Figure 4:**
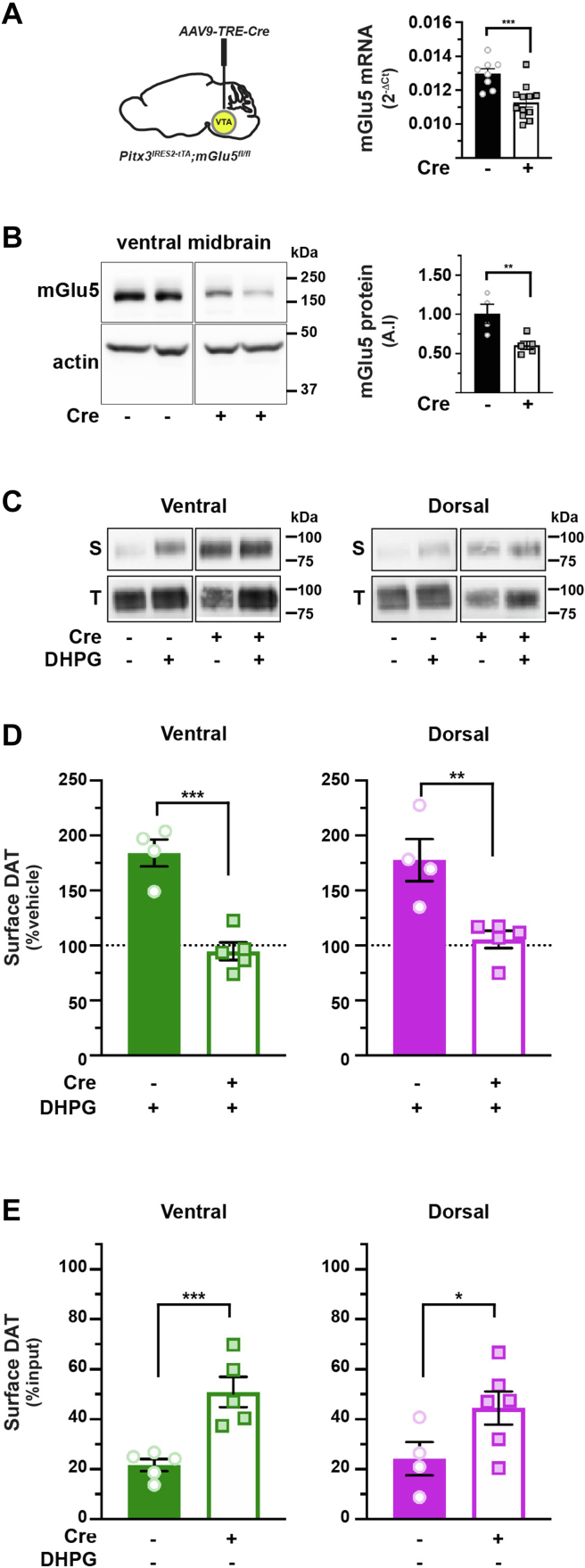
**mGlu5-mediated DAT trafficking is mediated presynaptically and impacts basal DAT surface expression**. *A* and *B*, *conditional mGlu5 silencing in DA neurons. Pitx3*^*IRES-tTA*^*;mGlu5*^*fl/fl*^ mouse VTA were bilaterally injected with either AAV9-TRE-eGFP (n = 7) or AAV9-TRE-Cre (n = 10). Midbrain tissue punches were obtained 4 to 5 weeks postinjection and assessed for either mGlu5 mRNA (*A*) or protein (*B*). *A*, *Left, viral injection schematic. Right, RT–qPCR* ∗∗∗*p* = 0.0005, one-tailed, unpaired Student’s *t* test, n = 8 (Cre) and 12 (eGFP). *B*, *midbrain mGlu5 protein levels. Left, representative immunoblot showing mGlu5 and actin signals* from *four independent mouse midbrains. Right, average data.* mGlu5 protein levels were normalized to actin loading controls. ∗∗*p* < 0.01, Student’s *t* test, n = 4 to 5. *C*–*E*, *Ex vivo striatal slice surface biotinylation. Pitx3*^*IRES-tTA*^*;mGlu5*^*fl/fl*^ mouse VTA were bilaterally injected with either AAV9-TRE-eGFP (n = 4) or AAV9-TRE-Cre (n = 5). Acute striatal slices were prepared from the indicated mice, treated ±10 μM DHPG (5 min, 37 °C), and DAT surface levels in ventral and dorsal striata were measured by slice biotinylation as described in Experimental procedures. *C*, *representative immunoblots:* surface (S) and total (T) DAT bands are presented for each of the indicated treatment conditions, in both ventral and dorsal striatum. *D*, *DHPG-stimulated DAT membrane insertion:* mean DAT surface levels are presented as %vehicle-treated contralateral hemisection. DAergic mGlu5 silencing significantly abolished DHPG-stimulated DAT insertion in both ventral (∗∗∗*p* = 0.0002) and dorsal (∗∗*p* = 0.003) striata, one-tailed, unpaired Student’s *t* test. *E*, *basal DAT surface expression:* mean surface DAT values are presented as %total DAT on the surface. DAergic mGlu5 silencing significantly increased basal DAT surface expression in ventral (∗∗∗*p* = 0.001, n = 5) dorsal (∗*p* = 0.035, n = 4–6) striata, one-tailed, unpaired Student’s *t* test. AAV, adeno-associated virus; DAT, dopamine transporter; DHPG, (*RS*)-3,5-dihydroxyphenylglycine; eGFP, enhanced GFP; qPCR, quantitative PCR; VTA, ventral tegmental area.

Given the viability of this approach, we tested whether DAergic mGlu5 is required for biphasic DAT trafficking. Bilateral AAV9-TRE-Cre injection into *Pitx3*^*IRES-tTA*^*;mGlu5*^*fl/fl*^ mouse VTA significantly reduced midbrain mGlu5 mRNA and protein expression as compared with AAV9-TRE-enhanced GFP (eGFP)-injected controls (Fig. 4, *A* and *B*). DAergic mGlu5 silencing completely abolished DHPG-stimulated DAT insertion in both VS and DS (Fig. 4, *C* and *D*), consistent with the premise that mGlu5 is expressed presynaptically in DA terminals, where it stimulates DAT trafficking in a cell-autonomous manner. We further hypothesized that DAT surface tone in the striatum is a balance between DRD2-stimulated DAT insertion and mGlu5-mediated retrieval and therefore predicted that mGlu5 silencing in DA neurons would lead to enhanced basal DAT surface expression. To test this possibility, we measured basal DAT surface levels following conditional mGlu5 silencing. DAergic mGlu5 loss significantly increased baseline DAT surface levels in both VS and DS, as compared with slices from control mice (Fig. 4*E*), suggesting that mGlu5-stimulated DAT retrieval significantly impacts striatal DAT surface tone.

### Biphasic mGlu5-stimulated DAT trafficking requires DRD2_auto_, retromer, and Rit2

**Figure 5:**
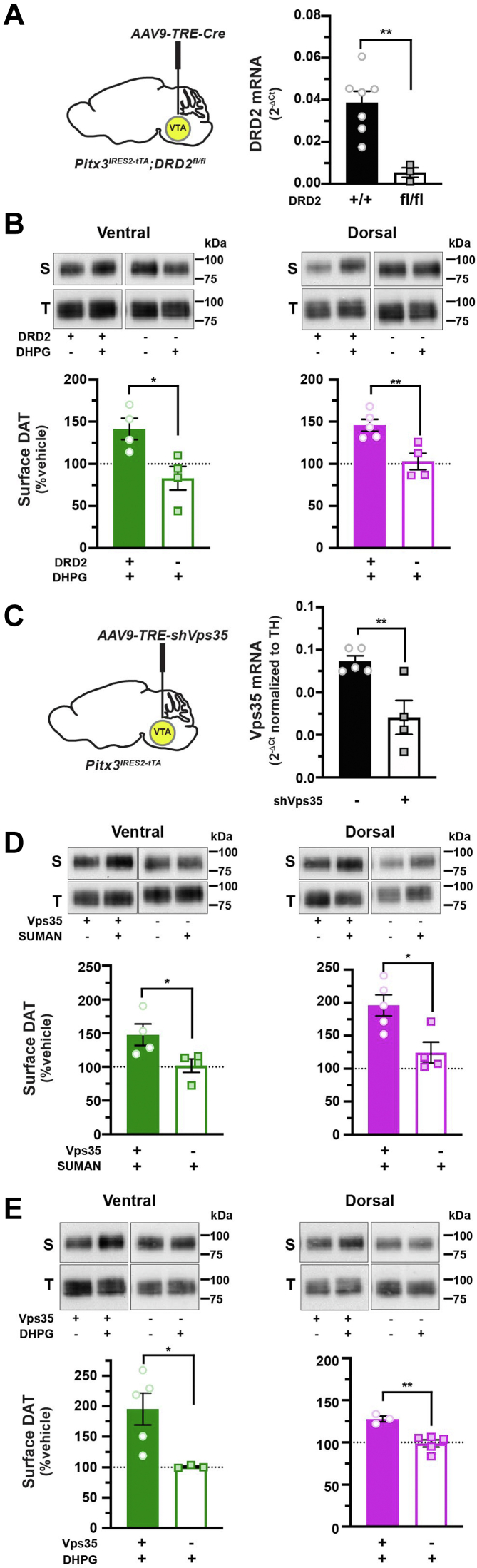
**mGlu5-mediated DAT insertion requires DRD2**_**auto**_**and intact retromer.***A*, *virally induced DRD2*_*auto*_*silencing. Left, Pitx3*^*IRES-tTA*^*;DRD2*^*fl/fl*^ mouse VTA were bilaterally injected with either AAV9-TRE-eGFP or AAV9-TRE-Cre. *Right*, midbrain *DRD2* mRNA levels were measured by RT–qPCR from tissue punches obtained 4 to 5 weeks postinjection. ∗∗*p* = 0.002, one-tailed, unpaired Student’s *t* test, n = 3 to 7. *B*, *ex vivo striatal slice surface biotinylation.* Acute striatal slices prepared from the indicated mice were treated ±10 μM DHPG (5 min, 37 °C), and DAT surface levels were measured by slice biotinylation as described in Experimental procedures. Representative immunoblots containing both surface (S) and total (T) DAT are shown above each graph, for all treatments. Average surface DAT values are expressed as % vehicle-treated contralateral hemisection ±SEM. DRD2_auto_ silencing abolished mGlu5-stimulated DAT membrane delivery in both ventral (∗*p* = 0.01) and dorsal (∗∗*p* = 0.004) striata, one-tailed, unpaired Student’s *t* test, n = 4 (ventral) and 4 to 5 (dorsal). *C*–*E*, *conditional Vps35 silencing in DA neurons.* (*C*, *left*) *Pitx3*^*IRES-tTA*^ mouse VTA were bilaterally injected with either AAV9-TRE-eGFP or AAV9-TRE-shVps35. *C*, *right*, midbrain Vps35 mRNA levels were measured by RT–qPCR from tissue punches obtained 4 to 5 weeks postinjection and were normalized to TH mRNA levels to account for varied DA neuron enrichment amongst tissue punches. ∗∗*p* = 0.005, one-tailed, unpaired Student’s *t* test, n = 4 to 5. *D* and *E*, *ex vivo striatal slice surface biotinylation.* Acute striatal slices were prepared from the indicated mice, treated with the indicated drugs (5 min, 37 °C), and DAT surface levels were measured by slice biotinylation as described inExperimental procedures. Surface DAT is expressed as % contralateral vehicle-treated hemisection ±SEM. *D*, *DRD2-stimulated DAT membrane delivery*: slices were treated ±170 nM sumanirole (SUMAN, 5 min, 37 °C). Vps35 silencing abolished DRD2-stimulated DAT membrane delivery in both ventral (∗*p* = 0.049) and dorsal (∗*p* = 0.02) striata, one-tailed, unpaired Student’s *t* test, n = 4 to 5. *E*, *mGlu5-stimulated DAT membrane delivery.* Slices were treated ±10 μM DHPG (5 min, 37 °C). Vps35 silencing abolished mGlu5-stimulated DAT membrane delivery in both ventral (∗*p* = 0.03) and dorsal (∗∗*p* = 0.003) striata, one-tailed, unpaired Student’s *t* test, n = 3 to 5. AAV, adeno-associated virus; DAT, dopamine transporter; DHPG, (RS)-3,5-dihydroxyphenylglycine; DRD2_auto_, DRD2 autoreceptor; eGFP, enhanced GFP; qPCR, quantitative PCR; TH, tyrosine hydroxylase; VTA, ventral tegmental area.

While hM3Dq-stimulated DAT insertion and retrieval are DRD2 and PKC dependent, respectively, it is unknown whether mGlu5-stimulated DAT trafficking is likewise dependent upon these mechanisms. It is further unclear whether DRD2-stimulated DAT insertion is mediated cell autonomously by the presynaptic DRD2_auto_ or by DRD2 activation elsewhere within the striatum, where DRD2s are widely expressed. To discriminate between these possibilities, we again leveraged our AAV-mediated Tet-OFF approach to conditionally excise DRD2_auto_ in a *Pitx3*^*IRES-tTA*^*;DRD2*^*fl/fl*^ mice and tested whether DRD2_auto_ was required for mGlu5-stimulated DAT insertion. Bilateral AAV9-TRE-Cre injection into *Pitx3*^*IRES-tTA*^*;DRD2*^*fl/fl*^ mouse VTA significantly decreased midbrain *DRD2* mRNA expression as compared with TRE-eGFP controls, consistent with DRD2_auto_ excision (Fig. 5*A*) but did not significantly alter basal DAT surface expression in either DS or VS (Fig. S2*D*). In VS and DS, DRD2_auto_ loss completely abolished DHPG-stimulated DAT insertion (Fig. 5*B*). Thus, DRD2_auto_ is specifically required for mGlu5-stimulated DAT insertion.

We next investigated the molecular mechanisms required for DRD2- and mGlu5-stimulated DAT insertion. We previously reported that internalized DAT targets to retromer/Vps35^+^ endosomes and that intact retromer complex is required for DAT delivery to the plasma membrane in DAergic neuroblastoma cell lines (28). We therefore hypothesized that retromer may likewise be required for both DRD2- and mGlu5-stimulated DAT surface delivery in DAergic terminals. To test this possibility, we conditionally silenced Vps35 in DA neurons *via* Tet-OFF AAV-mediated delivery of a validated mouse-directed Vps35 shRNA (53) and tested the ability of either sumanirole or DHPG to induce DAT insertion in either DS or VS DAergic terminals. AAV9-TRE-shVps35 significantly diminished *Vps35* mRNA expression in *Pitx3*^*IRES-tTA*^ mouse midbrain, as compared with TRE-eGFP-injected control mice (Fig. 5*C*) but did not significantly impact basal DAT surface expression in either DS or VS (Fig. S2*E*). DRD2- (Fig. 5*D*) and mGlu5-stimulated (Fig. 5*E*) DAT insertion in both VS and DS were significantly attenuated following DAergic Vps35 knockdown, as compared with slices from control-injected mice, demonstrating that retromer is required for both DRD2- and mGlu5-stimulated DAT insertion in DAergic terminals.

**Figure 6:**
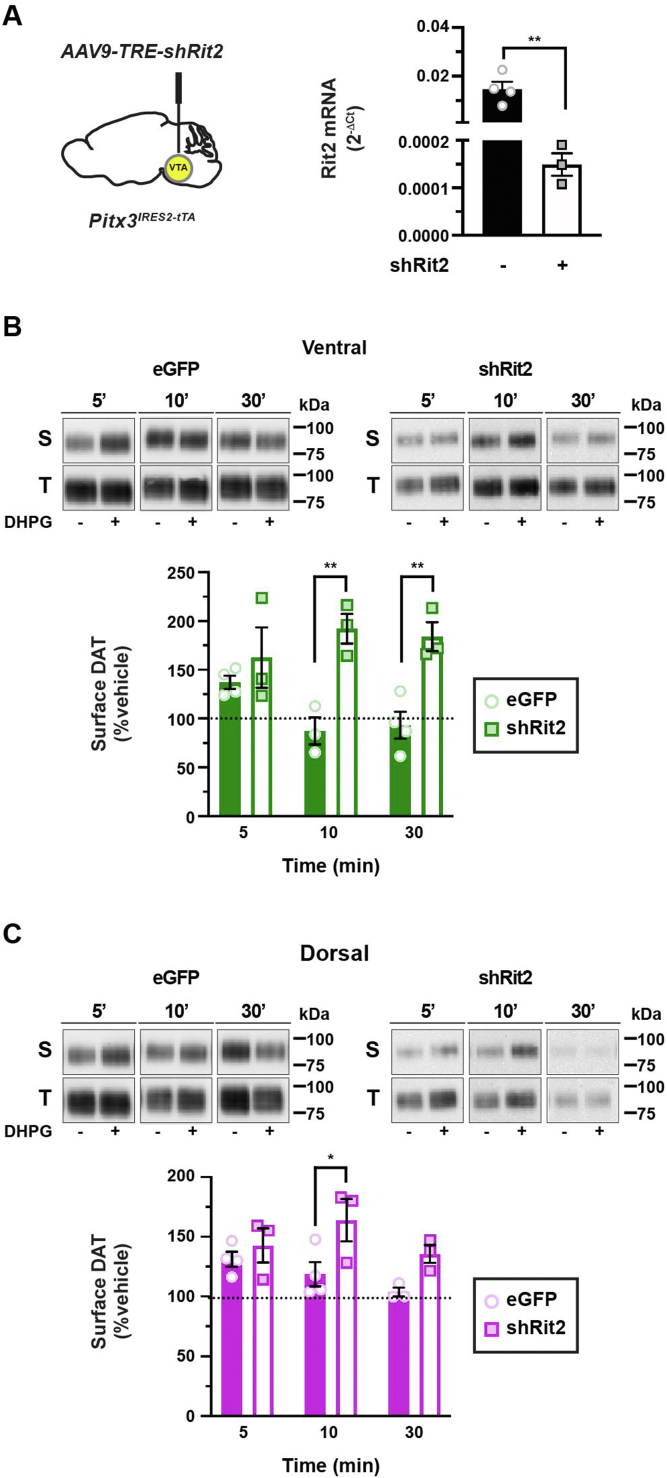
**Rit2 is required for mGlu5-mediated DAT retrieval, but not insertion.***A*, *conditional Rit2 silencing in DA neurons. Left, Pitx3*^*IRES-tTA*^ mouse VTA were bilaterally injected with either AAV9-TRE-eGFP or AAV9-TRE-Cre. *Right*, midbrain *Rit2* mRNA levels were measured by RT–qPCR from tissue punches obtained 4 to 5 weeks postinjection. ∗∗*p* = 0.005, one-tailed, unpaired Student’s *t* test, n = 3 to 4. *B* and *C*, *ex vivo striatal slice surface biotinylation.* Acute striatal slices were prepared from the indicated mice, treated ±10 μM DHPG for the indicated times (37 °C), and DAT surface levels were measured by slice biotinylation as described in Experimental procedures. Surface DAT is expressed as % contralateral vehicle-treated hemisection ±SEM. *B*, *ventral striatum*, two-way ANOVA: interaction: *F*_(2,14)_ = 3.35, *p* = 0.065; time: *F*_(2,14)_ = 0.29, *p* = 0.75, virus: *F*_(1,14)_ = 30.00, ∗∗∗∗*p* < 0.0001. Rit2 silencing significantly abolished DAT retrieval at 10 min (∗∗*p* = 0.002) and 30 min (∗∗*p* = 0.004), Sidak’s multiple comparisons test, n = 3 to 4. *C*, *dorsal striatum*, two-way ANOVA: interaction: *F*_(2,14)_ = 1.30, *p* = 0.30; time: *F*_(2,14)_ = 2.19, *p* = 0.15, virus: *F*_(1,14)_ = 11.44, ∗∗*p* = 0.004. Rit2 silencing significantly abolished DAT retrieval at 10 min (∗*p* = 0.02), Sidak’s multiple comparisons test, n = 3 to 4. DA, dopamine; DAT, dopamine transporter; DHPG, (*RS*)-3,5-dihydroxyphenylglycine; eGFP, enhanced GFP; qPCR, quantitative PCR; VTA, ventral tegmental area.

We previously reported that the neuronal GTPase Rit2 is required for phorbol ester–stimulated DAT endocytosis in cell lines and striatal slices (27, 29). However, it is unknown whether Rit2 is required for PKC-dependent DAT retrieval following mGlu5-stimulated DAT surface delivery. We predicted that, in the absence of Rit2, mGlu5 activation would still drive DAT surface delivery, but DAT would be unable to be subsequently retrieved. To test this possibility, we again leveraged the Tet-OFF approach to conditionally silenced DAergic Rit2 in *Pitx3*^*IRES-tTA*^ mice (29, 32) and tested whether Rit2 was required for either mGlu5-stimulated DAT insertion or retrieval. As previously reported, AAV9-TRE-shRit2 significantly reduced midbrain Rit2 mRNA (32) (Fig. 6*A*). DAergic Rit2 knockdown had no significant effect on mGlu5-stimulated DAT insertion in either VS (Fig. 6*B*) or DS (Fig. 6*C*) as compared with slices from TRE-eGFP-injected control mice. However, DAergic Rit2 silencing significantly blocked DAT retrieval and return to baseline in both VS and DS (Fig. 6, *B* and *C*), indicating that DAergic Rit2 is required for DAT retrieval following Gq-stimulated insertion in both DS and VS but not for insertion.

### DRD2-stimulated DAT insertion and presynaptic mGlu5 expression impact striatal DA dynamics

**Figure 7:**
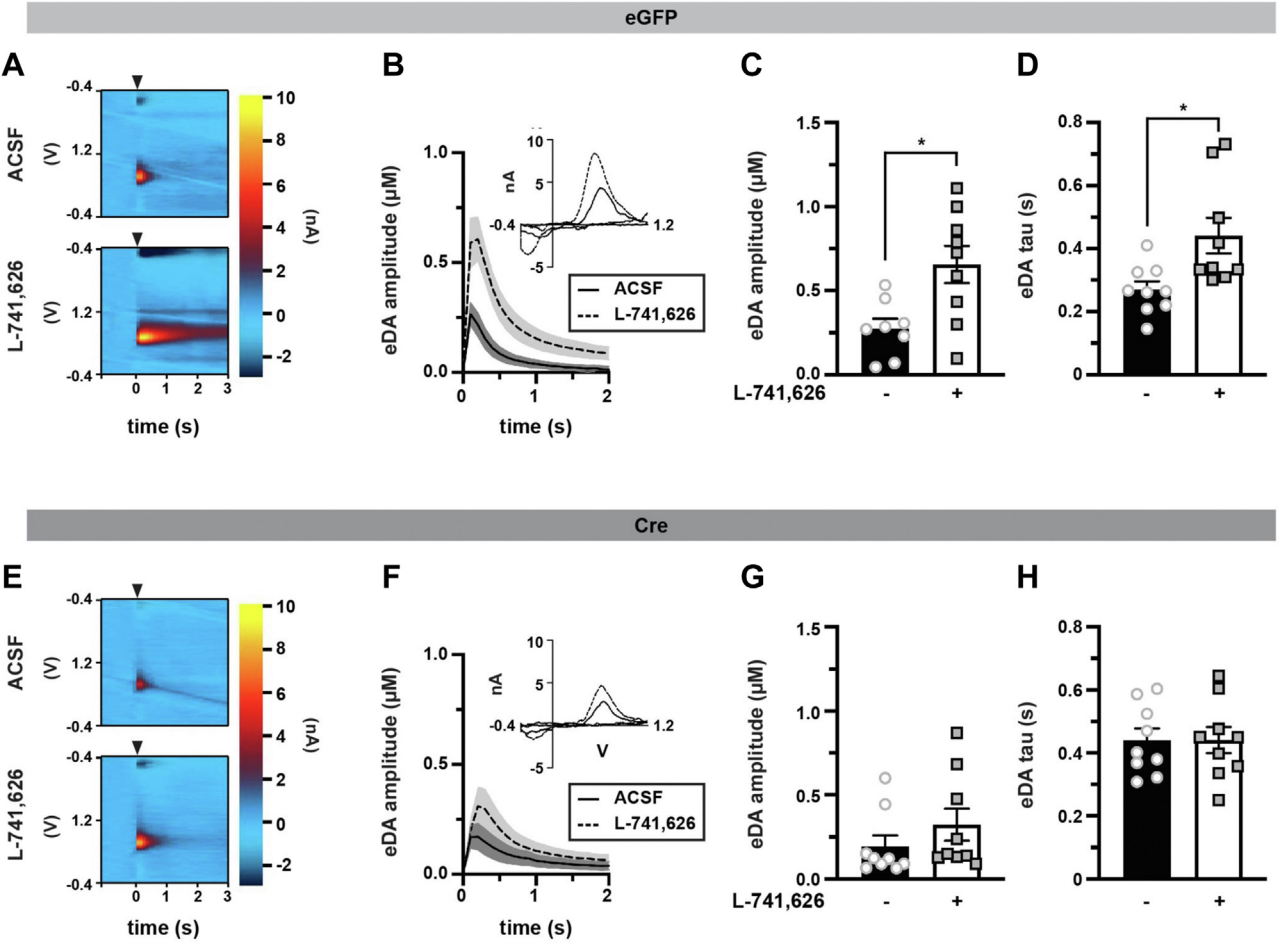
**DRD2-stimulated DAT insertion and mGlu5-mediated DAT retrieval impact DA release and clearance in dorsal striatum**. *Ex vivo fast-scan cyclic voltammetry: Pitx3*^*IRES-tTA*^*;mGlu5*^*fl/f*^ mouse VTA were bilaterally injected with either AAV9-TRE-eGFP (n = 7–9) or AAV9-TRE-Cre (n = 9). Acute striatal slices were prepared from the indicated mice, and electrically evoked DA transients were measured in dorsal striatum by FSCV as described in *Experimental procedures*. *A*–*D*, *eGFP:* (*A*) *Representative voltammograms:* voltammograms displaying evoked current over voltage cycles and time, in slices from eGFP-injected in the presence of ACSF alone (*top*) or supplemented with 25 nM L-741,626 (*bottom*). *Arrowheads* indicated delivery of single and squared wave pulse. *B*, *dopamine transients*, evoked DA transients in slices from eGFP-injected mice, treated ±L-741,626 (25 nM). Mean traces are presented with SEM indicated by *shaded areas*. *C*, *mean amplitudes:* mean amplitudes are presented in μM ±SEM. ∗Significantly greater than in ACSF alone, *p* = 0.02. *D*, mean decay tau, presented in seconds ±S.E.M. ∗Significantly longer clearance time than in ACSF alone, *p* = 0.03. *E*–*H*, *Cre:* (*E*) *representative voltammograms:* Voltammograms displaying evoked current over voltage cycles and time, in slices from eGFP-injected in the presence of ACSF alone (*top*) or supplemented with 25 nM L-741,626 (*bottom*). *Arrowheads* indicated delivery of single squared wave pulse. *F*, *dopamine transients:* evoked DA transients in slices from Cre-injected mice, treated ±L-741,626 (25 nM). Average traces are presented with SEM indicated by *shaded areas*. *G*, *mean amplitudes:* mean amplitudes are presented in micrometer ±SEM. DRD2 inhibition did not significantly alter DA release inhibition, *p* = 0.71. *H*, mean decay tau, presented in seconds ±SEM. DRD2 inhibition had no significant effect on DA clearance, *p* > 0.999. See Table 1 for all descriptive statistical analyses. ACSF, artificial cerebrospinal fluid; DA, dopamine; DAT, dopamine transporter; DRD2, D2 DA receptor; eGFP, enhanced GFP; VTA, ventral tegmental area.

Despite decades-long research that demonstrate regulated DAT trafficking, it remains unknown whether DAT trafficking impacts DAergic signaling. To test this possibility, we used fast-scan cyclic voltammetry (FSCV) in *ex vivo* dorsal striatal slices to ask whether (1) DRD2-mediated DAT insertion or (2) mGlu5 modulation of DAT basal surface levels were accompanied by changes in DA release and/or clearance. To this end, we conditionally excised mGlu5 selectively from DA neurons in *Pitx3*^*IRES-tTA*^*;mGlu5*^*fl/fl*^ mice injected with either AAV9-TRE-eGFP (control) or AAV9-TRE-Cre. We first probed how DRD2 activation impacts DA clearance in slices from control (eGFP) mice (Fig. 7, *A*–*D*). We predicted that evoked DA release would drive DRD2-stimulated DAT insertion and would be accompanied by decreased DA clearance times in slices from control mice. Thus, we compared DA transients evoked in artificial cerebrospinal fluid (ACSF) alone to those evoked in the presence of the DRD2-specific antagonist L-741,626 in separate slices (Fig. 7, *A*–*D* and Table 1). Electrically evoked DA transients in the presence of L-741,626 (25 nM) had an average amplitude of 655.6 ± 110.6 nM (Fig. 7*C*), and those recorded in ACSF alone were significantly smaller (361.1 ± 105.5 nM, Fig. 7*C*). DA clearance, measured as the exponential decay tau, was 0.44 ± 0.06 s in the presence of L-741,626 (Fig. 7*D*). However, the decay tau was significantly shortened when recorded in ACSF alone (0.27 ± 0.02 s; Fig. 7*D*). The differences in amplitude and tau were apparent from the first stimulation during baseline acquisition and were not induced by the stimulation protocol (Fig. S3, *A* and *B*).

**Table 1:** Effect of DRD2 inhibition and DAergic mGlu5 silencing on evoked DA transients

L-741,626	(−)	(+)
Amplitude (μM) (nM)		
*eGFP*	274.1 ± 167.7	655.6 ± 331.9^a^
*Cre*	194.9 ± 192.0	322.9 ± 285.5^b^
Tau (s)		
*eGFP*	0.27 ± 0.08	0.44 ± 0.17^c^
*Cre*	0.44 ± 0.11^d^	0.44 ± 0.13

Values are presented as average ± SD.

a *Amplitude:* Two-way ANOVA: interaction: *F*_(1,31)_ = 2.15, *p* = 0.15; virus: *F*_(1,31)_ = 5.67, *p* = 0.02, drug: *F*_(1,31)_ = 8.67, *p* = 0.006. Tukey’s multiple comparison test, n = 8 to 9. Significantly increased as compared with eGFP_(−L-741,626)_ slices, *p* = 0.02.

b *Amplitude:* Two-way ANOVA: interaction: *F*_(1,31)_ = 2.15, *p* = 0.15; virus: *F*_(1,31)_ = 5.67, *p* = 0.02, drug: *F*_(1,31)_ = 8.67, *p* = 0.006. Tukey’s multiple comparison test, n = 8 to 9. Significantly decreased as compared with eGFP _(+L-741,626)_ slices, *p* = 0.04.

c *Tau:* Two-way ANOVA: interaction: *F*_(1,32)_ = 4.20, *p* = 0.048; virus: *F*_(1,32)_ = 4.15; *p* = 0.05, drug: *F*_(1,32)_ = 4.21, *p* = 0.048. Tukey’s multiple comparison test, n = 9. Significantly increased as compared with eGFP_(−L-741,626)_ slices, *p* = 0.03.

d *Tau:* Two-way ANOVA: interaction: *F*_(1,32)_ = 4.20, *p* = 0.048; virus: *F*_(1,32)_ = 4.15; *p* = 0.05, drug: *F*_(1,32)_ = 4.21, *p* = 0.048. Tukey’s multiple comparison test, n = 9. Significantly increased as compared with eGFP_(−L-741,626)_ slices, *p* = 0.03.

We further tested whether the enhanced DAT surface expression that occurred in response to DAergic mGlu5 silencing (as shown in Fig. 4) impacted DA release and/or clearance. We predicted that since DAergic mGlu5 silencing increased DAT surface levels, DA clearance rates would be significantly faster than in slices from control mice. We in addition predicted that DRD2 activation would either further increase DA clearance or would not impact clearance because of potential ceiling effects on DAT membrane insertion. Results are shown in Figure 7, *E*–*H* and also Table 1. To our surprise, following presynaptic mGlu5 silencing, DRD2 inhibition had no effect on the average DA transient amplitude as compared with those acquired in ACSF alone (Fig. 7*G*), and DA amplitudes measured in ACSF alone were not significantly different in slices from eGFP- *versus* Cre-injected mice (Table 1). Similarly, DRD2 inhibition had no significant effect on decay tau values following DAergic mGlu5 silencing (Fig. 7*H*, *p* > 0.999). Moreover, despite increased DAT surface expression in quiescent slices, decay tau values in Cre-injected mice were significantly longer than those in eGFP-injected mice (Table 1, *p* = 0.03), rather than shorter. These unexpected results could not be explained by alterations in DAergic tone, as DAergic mGlu5 silencing did not significantly affect either DAT or TH protein levels in either VS (Fig. S3*C*) or DS (Fig. S3*D*). Moreover, DAergic mGlu5 silencing did not affect pSer40-TH in either striatal region (Fig. S3, *C* and *D*), suggesting that TH activity is properly regulated in the absence of mGlu5.

### mGlu5-stimulated DAT trafficking is required for motor learning

**Figure 8:**
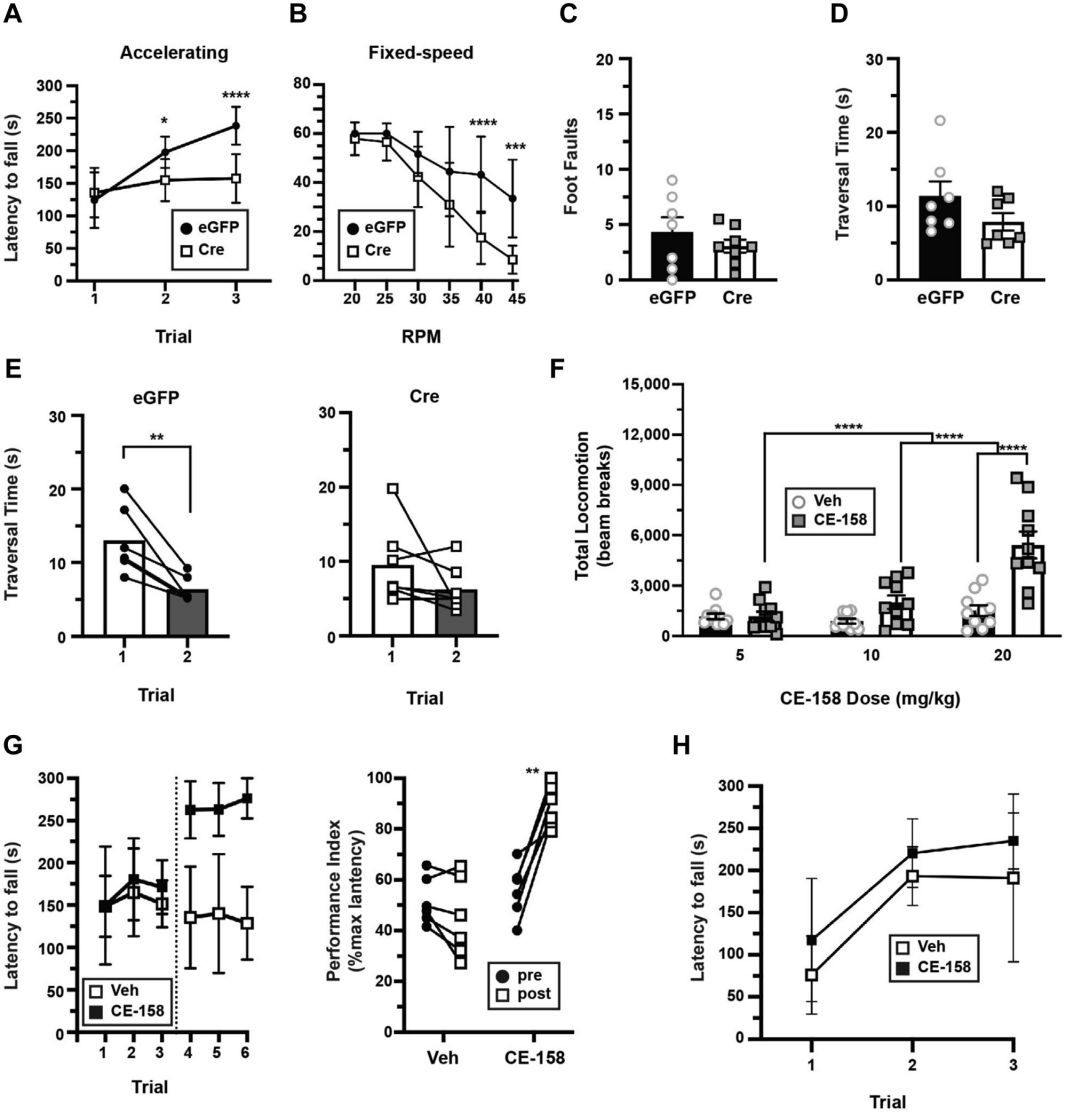
**DAergic mGlu5 is required for motor learning in a DAT-dependent manner**. *Pitx3*^*IRES-tTA*^*;mGlu5*^*fl/fl*^ mouse VTA were bilaterally injected with either AAV9-TRE-eGFP (n = 7–9) or AAV9-TRE-Cre (n = 9) and assessed by the indicated behavioral assays. *A*, *accelerating rotarod:* Mice were assessed over three consecutive trials as described in Experimental procedures and are presented as latency to fall (seconds) in each trial, ±SD. Two-way repeated-measures ANOVA: trial × virus: (*F*_(2,30)_ = 17.04, ∗∗∗∗*p* < 0.0001; trial: *F*_(2,30)_ = 38.22, ∗∗∗∗*p* < 0.0001, virus: *F*_(1,15)_ = 6.65, ∗*p* = 0.02, subject: *F*_(15,30)_ = 5.02, ∗∗∗∗*p* < 0.0001). mGlu5 silencing in DA neurons significantly dampened performance on trials 2 (∗*p* = 0.049) and 3 (∗∗∗∗*p* < 0.0001), Bonferroni’s multiple comparisons test. *B*, *fixed speed rotarod:* Mice were assessed over the indicated consecutive speeds as described in Experimental procedures and are presented as latency to fall (seconds) at each speed tested, ±SD. Two-way repeated-measures ANOVA: speed × virus: *F*_(5,75)_ = 5.06, ∗∗∗∗*p* < 0.0005; speed: *F*_(5,75)_ = 44.91, ∗∗∗∗*p* < 0.0001, virus: *F*_(1,15)_ = 12.23, ∗∗*p* = 0.003, subject: *F*_(15,15)_ = 4.09, ∗∗∗∗*p* < 0.0001. mGlu5 silencing in DA neurons significantly dampened performance at 40 (∗∗∗∗*p* < 0.0001) and 45 rpm (∗∗∗*p* = 0.0002), Bonferroni’s multiple comparisons test. *C*–*E*, *challenge balance beam.* Mean foot fault numbers (*C*) and beam traversal time (seconds) (*D*) are presented. mGlu5 silencing in DA neurons did not significantly affect either foot fault number (*p* = 0.78) or mean traversal times (*p* = 0.15), two-tailed, unpaired Student’s *t* test. *E*, *traversal time improvement.* eGFP (*control*, *left*) mouse traversal times significantly improved between trials 1 and 2 (∗*p* = 0.02), whereas mice injected with Cre (*right*) failed to improve their performance (*p* = 0.77), two-tailed, paired Student’s *t* test. *F*, *dose-dependent effect of CE-158 on horizontal locomotion in wildtype mice.* Wildtype mice were habituated to photobeam activity chambers for 45 min and were injected (I.P.) with either vehicle (on day 1) or the indicated CE-158 dose (on day 2) and their horizontal locomotion was measured for 90 min. Cumulative locomotion is presented as total postinjection beam breaks. Two-way ANOVA: dose × drug: *F*_(2,54)_ = 12.01, ∗∗∗∗*p* < 0.0001; dose: *F*_(2,54)_ = 18.70, ∗∗∗∗*p* < 0.0001, drug: *F*_(1,54)_ = 25.36, ∗∗∗∗*p* < 0.0001. 20 mg/kg CE-158 significantly increased locomotion as compared with vehicle (∗∗∗∗*p* < 0.0001), 10 mg/kg (∗∗∗∗*p* < 0.0001), and 5 mg/kg (∗∗∗∗*p* < 0.0001), Bonferroni’s multiple comparisons test, n = 9 to 10. *G*, *accelerating rotarod rescue studies with CE-158:* mice with DAergic mGlu5 silencing were assessed over three trials, injected I.P. with either saline or CE-158 (10 mg/kg), and reassessed over three trials 15 min postinjection. *Left*, raw rotarod results presented as latency to fall (seconds) in each trial, ±SD. *Right*, rotarod performance indices, expressed as %maximal latency to fall. Mice injected with CE-158 performed significantly better postinjection as compared with preinjection (∗∗*p* = 0.0014), whereas saline-injected mice did not perform significantly better (*p* = 0.11). Two-tailed paired *t* test, n = 6. *H*, *effect of CE-158 on wildtype mouse performance on accelerating rotarod.* Wildtype mice were injected with either saline or CE-158 (10 mg/kg) and assessed on the accelerating rotarod. Data are presented as latency to fall (seconds) in each trial, ±SD. CE-158 had no significant effect on mouse performance over three trials. Two-way ANOVA: trial × drug: *F*_(2,22)_ = 0.085, *p* = 0.92; trial: *F*_(2,22)_ = 18.17, *p* < 0.0001, drug: *F*_(1,11)_ = 3.136, *p* = 0.11, n = 6 (vehicle) or 7 (CE-158). AAV, adeno-associated virus; DA, dopamine; DAT, dopamine transporter; eGFP, enhanced GFP; VTA, ventral tegmental area.

In light of our new understanding that DAT undergoes biphasic surface trafficking in DAergic terminals, we next aimed to test whether disrupting mGlu5-mediated and biphasic DAT trafficking impacted DA-dependent motor behaviors. *Pitx3*^*IRES-tTA*^*;mGlu5*^*fl/fl*^ mouse VTA were injected with either AAV9-TRE-Cre or TRE-eGFP, and mice were assessed across a battery of locomotor assays. mGlu5 loss from DA neurons did not significantly affect baseline horizontal (Fig. S4, *A* and *B*), vertical (Fig. S4*C*), or fine (Fig. S4*D*) locomotion. However, DAergic mGlu5 silencing significantly impaired motor learning on the accelerating rotarod as compared with control-injected mice (Fig. 8*A*) and also significantly decreased performance on the fixed speed rotarod (Fig. 8*B*). DAergic mGlu5 silencing did not significantly impact performance on the challenge balance beam, measured as both number of foot faults (Fig. 8*C*) and average traversal time (Fig. 8*D*). In addition, we noted that control mice traverse the beam significantly faster in trial 2 *versus* trial 1 and that DAergic mGlu5 is required for this improved performance (Fig. 8*E*). We further tested whether decreased rotarod performance was due to either muscle weakness/fatigue or gait disturbance, using the grip strength assay and gait assessment assays, respectively. DAergic mGlu5 silencing caused a modest, but significant, decrease in grip strength (Fig. S4*E*) but had no impact on gait as compared with control-injected mice, as measured by stride length, stride width, and toe spread, in both forelimbs and hindlimbs (Fig. S5).

Motor learning requires DA (54). Given that DAergic mGlu5 silencing significantly increased basal DAT surface levels, we hypothesized that enhanced DAT surface expression would, in parallel, enhance DA clearance from the extracellular space and limit the extracellular DA necessary for motor learning. Alternatively, DAergic mGlu5 could play a role in motor learning that is independent of DAT trafficking. If enhanced DAT surface expression and function was solely responsible for rotarod deficits in mice with DAergic mGlu5 silencing, we predicted that partially blocking DAT with a subthreshold dose of a DAT-specific inhibitor would rescue the phenotype. A recently reported DAT inhibitor, (S,S)-CE-158, is highly selective for DAT over the norepinephrine transporter, permeates the blood–brain barrier, and increases extracellular DA levels in striatum following I.P. injection in rats (55). We first tested which *in vivo* CE-158 doses are required to block DAT and drive hyperlocomotion in mice, a hallmark of enhanced extracellular DA. Wildtype mice were injected (I.P.) with either 5, 10, or 20 mg/kg CE-158, and horizontal locomotion was measured. Only the 20 mg/kg CE-158 dose resulted in significantly increased locomotion as compared to either vehicle, 5 mg/kg, or 10 mg/kg injections (Figs. 8*F* and S6). We therefore tested whether the subthreshold 10 mg/kg CE-158 dose could rescue motor learning following DAergic mGlu5 silencing. Mice were initially assessed on the rotarod, injected with either vehicle or CE-158 (10 mg/kg, I.P.), and then reassessed 15 min postinjection. CE-158 significantly improved mouse performance on the accelerating rotarod, whereas vehicle injection had no significant effect on performance (Fig. 8*G*). Importantly, 10 mg/kg CE-158 (I.P.) had no impact on wildtype mouse performance on the accelerating rotarod (Fig. 8*H*). Thus, CE-158 does not impact motor learning *per se* but selectively rescues motor learning following DAergic mGlu5 silencing, consistent with a potential role for mGlu5-stimulated DAT trafficking in motor learning.

## Discussion

**Figure 9:**
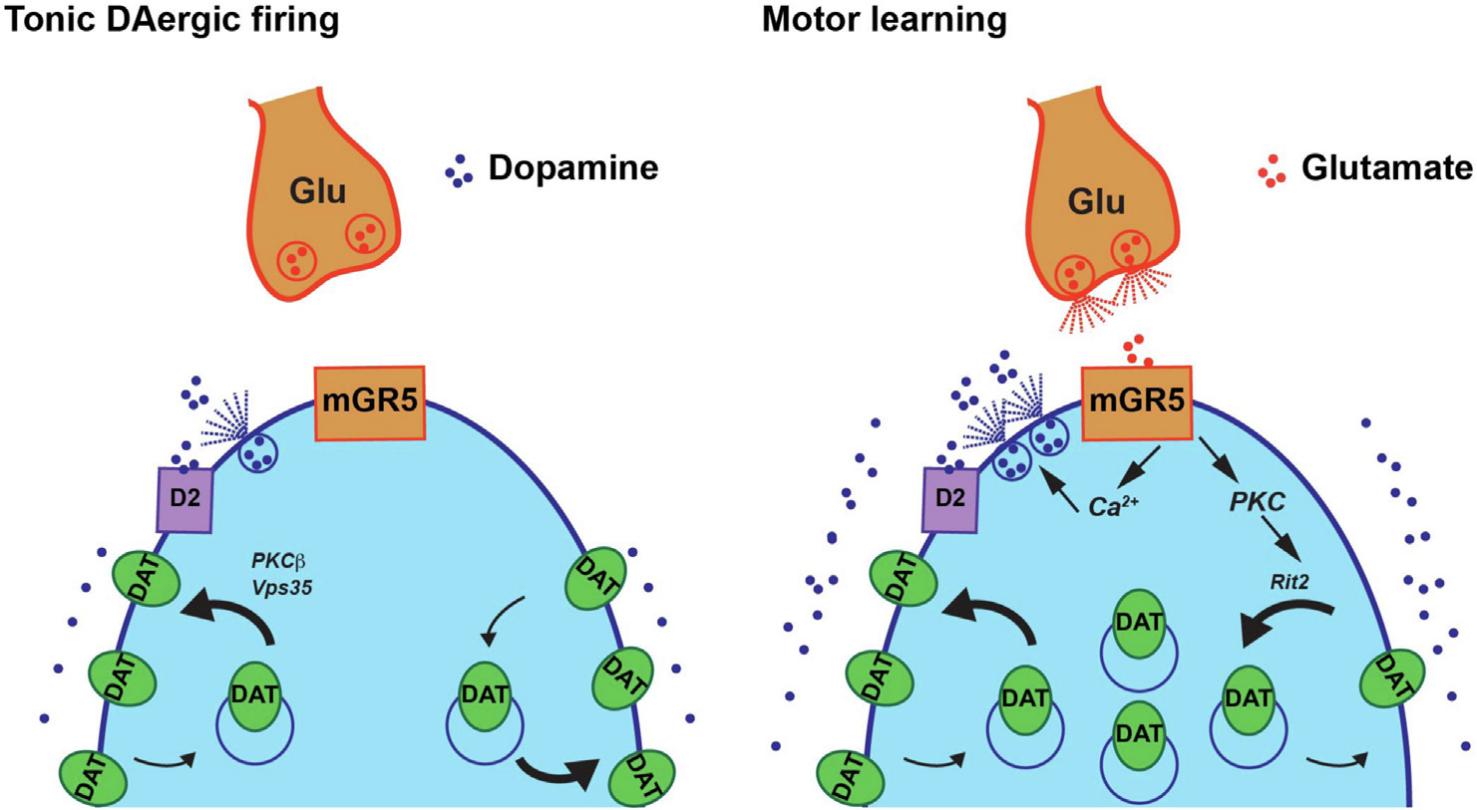
**Striatal DAT trafficking model.** A presynaptic DAergic terminal and a glutamatergic afferent are depicted. *Left*, *tonic DA neuron firing*. During tonic DA release, DRD2_auto_ activity robustly delivers DAT to the plasma membrane in a PKCβ- and Vps35-dependent manner. Dense DAT surface expression restricts extracellular DA levels. *Right, glutamate release during motor learning.* When DA demand is higher, such as during motor learning, glutamate release from striatal glutamatergic afferents activates presynaptic mGlu5 (mGR5), which elevates intracellular calcium (Ca^2+^) and enhances DA release and DAT membrane insertion. However, mGlu5-mediated PKC activation concurrently drives DAT membrane retrieval in a Rit2-dependent manner. DAT retrieval decreases DAT surface levels, relative to tonic firing, to sustain extracellular DA levels during a period of increased demand. DA, dopamine; DAT, dopamine transporter; DRD2_auto,_ DRD2 autoreceptor.

Biphasic DAT trafficking has the potential to mold the DA signal during the ebb and flow of plastic events throughout the striatum. Our results point to a previously unknown role for striatal glutamatergic signaling to temporally shape DA signaling *via* DAT trafficking. Our emerging model is depicted in Figure 9. We hypothesize that under basal conditions, in which DA neurons fire tonically, DRD2_auto_ activation delivers DAT to the plasma membrane in a PKCβ- and retromer-dependent manner. If DA demand is higher, such as during motor learning, glutamate release within the striatum activates mGlu5 on DAergic terminals, reducing surface DAT, and thereby enhancing the half-life of extracellular DA. This simple model does not take into account the plethora of presynaptic GPCRs, including group II and III mGlus, which also likely influence DAT surface expression. However, given the ability of DAT to rapidly traffic, it appears certain that presynaptic receptor signals continually integrate to orchestrate DAT surface levels and, ultimately, the extracellular DA signal.

Our results reveal that DAT traffics biphasically in response to presynaptic Gq receptor activation, *via* either hM3Dq (Figs. 1 and 2) or the presynaptic mGlu5 (Figs. 3 and 4). These results were initially surprising in light of copious reports by our laboratory (24, 25, 27, 28, 29, 33, 56, 57) and others (26, 58, 59, 60) that demonstrated direct PKC activation decreases DAT surface expression in cell lines and striatal slices. Moreover, coexpressing DAT and the Gq-coupled neurokinin receptor decreased DAT surface expression in a PKC-dependent manner in cell lines (35). A more recent study similarly demonstrated that the Gq-coupled muscarinic receptors, M1 and M5, stimulate DAT internalization in primary DA neuronal culture and in mouse midbrain (34). Interestingly, our results indicated that PKC-dependent DAT internalization does indeed occur. However, in the conditions we tested, it appears that in the striatum, PKC-dependent DAT internalization functions as a retrieval mechanism to temper DAT surface levels following stimulated DAT membrane insertion.

Our findings demonstrated that the initial Gq-stimulated DAT insertion requires DA release and DRD2 activation. Previous studies in synaptosomes and transfected cell lines reported that DAT function and surface expression rapidly increase in response to treatment with quinpirole (39, 61), a nonselective DRD2/DRD3 agonist. Moreover, DRD3 activation was sufficient to increase DAT surface expression in non-neuronal cell lines cotransfected with DRD3 and DAT (62). Both DRD2 and DRD3 are expressed in the striatum (63), and heretofore, it was not known whether quinpirole-stimulated DAT trafficking in terminals was mediated by DRD2, DRD3, or both. Moreover, it is also unknown whether DRD2 activation impacts DAT directly in presynaptic terminals or *via* indirect means within the striatum where DRD2 is widely expressed and modulates both glutamate (64, 65, 66) and acetylcholine (67) release. Given that Gq-stimulated DAT insertion selectively requires presynaptic DRD2_auto_, our results suggest that (1) presynaptic DRD2 activation is sufficient and required for Gq-stimulated DAT insertion and (2) DRD2 activation elsewhere within the striatum does not contribute to DAT membrane insertion in response to Gq-evoked DA release. Surprisingly, depleting DA stores *in vivo* with reserpine increased basal DAT surface expression in the DS but not VS (Fig. S1*A*). Since reserpine blocks the vesicular monoamine transport, and therefore depletes all vesicular monoamine stores, this result raises the possibility that there may be differential DAT regulation by either norepinephrine or 5-hydroxytryptamine in DS but not VS.

Increasing evidence suggests that DA homeostasis and DAT regulation may differ regionally in VS *versus* DS (68). We also observed region-specific differences for DRD2- and Gq-stimulated DAT trafficking. Direct DRD2 activation with sumanirole was sufficient for DAT membrane insertion in both DS and VS (Fig. S1). However, DAT remained elevated at the plasma membrane following DRD2-stimulated membrane insertion in the DS, whereas DAT returned to baseline in the VS, despite lack of any exogenous Gq receptor activation (Fig. S1*B*). Similarly, following hM3Dq-stimulated DAT insertion, we observed differential kinetics for return to baseline in VS *versus* DS, with significantly faster retrieval in VS *versus* DS (Fig. 1); however, this region-specific difference did not occur in response to mGlu5 activation (Fig. 3). It is possible that differential DRD2 expression in VS *versus* DS could contribute to these regional differences, but it is currently not known whether such differences exist. There may also be differential spontaneous glutamate release from glutamatergic terminals in VS *versus* DS slices, which would drive DAT retrieval *via* mGlu5 in VS preferentially to DS. A large population of mesolimbic, but not nigrostriatal, DA neurons corelease glutamate (69, 70), so it is also possible that glutamate corelease from DA terminals may play an autocrine regulatory role in VS *versus* DS. Another possible contributor to striatal DAT trafficking could arise from striatal cholinergic interneurons signaling *via* the Gq-coupled M5 receptor, which drives DAT internalization in midbrain (34) and is reportedly expressed in striatal DAergic terminals where it potentiates DA signaling (71). Future studies will explore how glutamatergic and cholinergic signaling converge onto DA terminals to influence DAT surface levels and DA signaling.

We previously reported that the neuronal, ras-like GTPase, Rit2, is required for PKC-stimulated DAT endocytosis in both cell lines (27, 29) and DAergic terminals (29) in response to phorbol ester treatment. We in addition found that endocytosed DAT primarily targets to retromer^+^ endosomes and that intact retromer complex is required for DAT membrane delivery in neuroblastoma cell lines (28). Here, we demonstrate that Vps35^+^ retromer complex is required for stimulated DAT membrane insertion (Fig. 5), and that Rit2 is required for DAT retrieval but not DAT insertion (Fig. 6). Consistent with our findings, a recent study found that DAT and Vps35 colocalize in axons of the medial forebrain bundle (72). Interestingly, apart from their roles in DAT trafficking (and other cellular processes), both Vps35 and Rit2 have been identified as PD risk factors. A Vps35 mutation (D620N) from late onset PD patients was recently reported (73), and a D620N-Vps35 mouse model exhibited reduced DAT surface expression and decreased DA clearance in *ex vivo* slices (74), consistent with disrupted and retromer-dependent DAT membrane delivery.

How does biphasic DAT trafficking impact DA signaling and/or DA-dependent behaviors? To begin to address this challenging question, we conditionally silenced mGlu5 in *Pitx3*^*IRES-tTA*^*;mGlu5*^*fl/fl*^ midbrain DA neurons. Although mGlu5 is expressed at high levels in the striatum, it was not known whether mGlu5 is expressed presynaptically in DAergic terminals. Conditional DAergic mGlu5 silencing completely abolished DHPG-stimulated DAT membrane insertion (Fig. 4, *C* and *D*), clearly demonstrating that mGlu5 is expressed in DA terminals, where it directly drives biphasic DAT trafficking. Furthermore, DAergic mGlu5 silencing increased basal DAT surface expression (Fig. 4*E*) in both VS and DS, suggesting that mGlu5 is specifically pivotal for setting DAT surface tone.

We explored the impact of both DRD2- and mGlu5-stimulated DAT trafficking on DA release and clearance in DS by FSCV. Electrically evoking DAT release in ACSF will also drive release of multiple striatal neurotransmitters close to the recording site, including acetylcholine, glutamate, and neuropeptides. Therefore, the tau measured under ACSF conditions likely reflects the summated DAT trafficking induced *via* multiple signaling pathways including DRD2-mediated insertion and mGlu5-mediated retrieval. In slices from control mice, DA clearance times were significantly longer in slices pretreated with the DRD2 antagonist L-741,626, than those measured in ACSF (Fig. 7*D*), consistent with DRD2-mediated increases in DAT surface levels. DAT is also subject to catalytic regulation both by post-translational modifications, such as phosphorylation and palmitoylation (20), and oligomerization (75). Thus, we cannot rule out that these mechanisms also contributed to changes in DA clearance in our preparations. Surprisingly, we observed DAT release differences between ACSF- and L-741,626-pretreated slices from the very first evoked DA transient (Fig. S2, *C* and *D*), suggesting that DRD2-mediated regulation had already occurred prior to initiating recording, either *in vivo* or after preparing slices, and was rescued by the L-741,626 pretreatment. Multiple previous studies indicate that DRD2 inhibition of DA release in *ex vivo* slices only occurs following higher frequency stimulation (76, 77, 78). However, these studies applied DRD2 antagonists only after driving inhibition of DA release, rather than comparing slices incubated with or without DRD2 antagonists, as in our study. This suggests that prepared slices may already have some degree of DRD2 regulation, and higher frequency train stimulus is required to achieve further DRD2-dependent suppression of DA release. In addition, most previous studies relied on sulpiride, which cannot distinguish between DRD2- and DRD3-mediated events, whereas L-741,626 is ∼50-fold more selective for DRD2 at the concentrations we used. Thus, DRD3 may contribute to DA release modulation.

We hypothesized that since conditional mGlu5 silencing increased basal DAT surface expression, this would translate into more rapid clearance rates in ACSF, as DAT would be inserted *via* DRD2 activation but would fail to be rapidly retrieved in the absence of presynaptic mGlu5. To our surprise, DAergic mGlu5 silencing significantly increased the tau to clear DA under basal conditions as compared with control mice (Table 1), which suggests a potential for suppressed intrinsic DAT function. Moreover, while DRD2 inhibition increased the clearance tau under basal conditions, it had no further effect on DA clearance rates in the absence of mGlu5 (Fig. 7*H* and Table 1), suggesting that mGlu5 may impact DRD2 function. Indeed, the DA transient amplitude was also unaffected by DRD2 inhibition following presynaptic mGlu5 silencing, as compared with controls (Fig. 7*G*), raising the possibility that mGlu5 deletion may impact DA release *in vivo*, either *via* modulating voltage-gated Ca^2+^ channels or *via* Kv1 channels, which are reported to influence DA release (79, 80). Alternatively, several reports indicate that DA receptors including DRD2 and DRD3 undergo PKC-stimulated internalization (81, 82). It is possible that, in addition to regulating DAT surface expression, mGlu5 may regulate DRD2 and/or DRD3 surface expression. Although our data suggest that DRD2 blockade alone is sufficient to block mGlu5-mediated DAT insertion, we cannot rule out that in the absence of mGlu5, there may be an aberrant DRD3 contribution that modulates DAT surface levels during evoked DA release. Indeed, DRD2 and DRD3 have been shown to differentially regulate DAT function (83). While we did observe increased DAT surface expression in quiescent slices, it is possible that other signaling cascades, activated by electrically evoked neurotransmitter release, may integrate to suppress DAT surface expression and/or intrinsic function, even in the absence of DAergic mGlu5.

Although DAergic mGlu5 silencing had little effect on baseline locomotor behavior, it significantly disrupted motor learning and coordination measured using the rotarod (Fig. 8, *A* and *B*). Consistent with our findings, a previous study that locally infused mGlu5-specific antagonists into the striatum found that striatal mGlu5 activity promotes accelerating rotarod performance (84). Although rotarod performance was profoundly impacted in our study, DAergic mGlu5 silencing had no overall effect on coordinated movement on the challenge balance beam (Fig. 8, *C*–*E*). However, improved challenge balance beam performance over consecutive trials on test day demonstrated by control mice was absent in DAergic mGlu5-silenced mice (Fig. 8). This suggests that although DAergic mGlu5 excision does not affect coordination *per se*, it may impact other unknown aspects of balance beam performance, such as learning and/or habituation on test day. In fact, global mGlu5 knockout mice also exhibit deficits in spatial learning memory and fear conditioning acquisition (85), both of which are DAergic-dependent learning behaviors, implicating mGlu5 and potentially DAT trafficking in broader learning behaviors.

We hypothesized that presynaptic mGlu5 silencing disrupts DAT retrieval, leading to increased surface DAT, faster *in vivo* clearance times, and insufficient extracellular DA to facilitate rotarod performance. Consistent with this hypothesis, conditional mGlu5 silencing significantly increased baseline DAT surface expression in VS and DS (Fig. 4*E*), and rotarod deficits were rescued by administering a subthreshold dose of the novel DAT-specific inhibitor, CE-158 (Fig. 8*G*). Given that conditional mGlu5 silencing had no effect on total DAT and TH protein levels in either VS or DS (Fig. S3, *B* and *C*), nor did it impact activated TH, as determined by quantifying pSer40-TH (Fig. S3, *B* and *C*), our results strongly suggest that DAT trafficking dysregulation contributed to the motor phenotypes we observed. However, there are several other factors that could underlie and/or contribute to the lack of motor learning following mGlu5 silencing. DAergic mGlu5 silencing could be inhibiting DA neuron excitability at the somatodendritic level and suppressing increased DA neuron firing rates. Consistent with this possibility, chemogenetic Gq-coupled receptor activation in VTA DA neurons was recently demonstrated to increase their firing rates (38), and activation of group I mGluRs in midbrain and basal ganglia nondopaminergic neurons likewise increased neuronal firing (86). However, although it was recently reported that group I mGluR activation depresses induced pluripotent stem cells in VTA DA neurons, it appears to do so *via* mGlu1, not mGlu5, suggesting that mGlu5 may not contribute to DA neuron excitability in VTA (87). Further studies that utilize *in vivo* voltametric or postsynaptic DA sensors would be pivotal for understanding DA dynamics during complex behaviors.

In summary, we found that Gq-coupled presynaptic receptors drive dynamic and biphasic DAT trafficking that differs in VS and DS and identified the specific presynaptic mechanisms that are required for regulated DAT trafficking *in situ.* Importantly, these studies are the first to demonstrate a cell-autonomous DAT trafficking mechanism driven by an endogenous Gq-coupled GPCR in intact DA terminals and suggest that glutamatergic signaling onto DAergic terminals may shape DAergic transmission *via* mGlu5-stimulated DAT trafficking and that DAT trafficking is integral to DAergic behaviors, such as motor learning.

## Experimental procedures

### Materials

CNO (4936), reserpine (2742), L-741,626 (1003), LY 333531 (ruboxistaurin; 4738), sumanirole maleate (2773), GF 109203X (BIM I), and DHPG (0342) were from Tocris. (S,S)-CE-158 was synthesized as previously described (55). All other reagents were from either Sigma–Aldrich or Fisher Scientific and were of the highest possible grade.

### Mice

All mouse studies were conducted in accordance with protocol #202100046 (formerly A-1506, H.E.M.), approved by the UMass Chan Medical School Institutional Animal Care and Use Committee. *Pitx3*^*IRES-tTA*^*/+* mice (on the C57Bl/6J background) were the generous gift of Dr Huaibin Cai (National Institute on Aging) and were continuously backcrossed to C57Bl/6J mice (Jackson Laboratories). *TRE-hM3Dq* (#014093), *Drd2*^*fl/fl*^ (#020631), and *mGlu5*^*fl/f*^ (#028626) mice, all on the C57Bl/6J background, were obtained from Jackson Laboratories and were backcrossed to either C57Bl/6J or to *Pitx3*^*IRES-tTA*^*/+* mice to generate *Pitx3*^*IRES-tTA*^;*TRE-hM3Dq*, *Pitx3*^*IRES-tTA*^;*Drd2*^*fl/fl*^, and *Pitx3*^*IRES-tTA*^;*mGlu5*^*fl/fl*^ mice, respectively. Mice were maintained in 12 h light/dark cycle (lights on at 0700) at constant temperature and humidity. Food and water were available ad libitum.

### AAVs and stereotaxic surgeries

#### AAVs

pscAAV-TRE3g-eGFP and pscAAV-TRE3g-miR33-shRit2-eGFP AAV9 particles were produced as described (32).

#### pscAAV-TRE3g-shVps35-eGFP and pscAAV-TRE3q-Cre-eGFP

pscAAV-TRE3g-miR33-shRit2-eGFP was digested with BglII and PstI as backbone. miR33-shVps35 insert was synthesized as a gene block with shRNA antisense sequence ATA ATC CAG AAC ATT ACT AAG (V2LMM_36638; Dharmacon), previously validated (53). Cre recombinase insert was PCR amplified with addition of 5′ BglII and 3′ PstI sites from pAAV-CB6-PI-Cre (UMass Viral Vector Core). High-titer AAV9 particles were produced by the University of Massachusetts Medical School Viral Vector Core as previously described (29, 32).

#### Survival surgeries

Mice aged 3 to 4 weeks were anesthetized with I.P, 100 mg/kg ketamine (Vedco, Inc) and 10 mg/kg xylazine (Akorn, Inc). About 20% mannitol (NeogenVet) was administered I.P. >15 min prior to viral delivery, to increase viral spread (88). Mice were prepared and placed in the stereotaxic frame (Stoelting, Inc). About 1 μl of the indicated viruses were administered bilaterally to the VTA (Bregman: anterior/posterior: −3.08 mm, medial/lateral: ±0.5 mm, dorsal/ventral −4.5 mm) at a rate of 0.2 μl/min. Syringes were left in place for a minimum of 5 min postinfusion prior to removal. Viral incubation was a minimum of 4 weeks. Viral expression was confirmed visually by the presence of GFP in the midbrain and/or by RT–quantitative PCR (qPCR).

### RNA extraction and RT–qPCR

RNA was isolated from mouse midbrain punches using RNAqueous-Micro Kit RNA isolation (Thermo Fisher Scientific). Ventral midbrain samples were bilaterally collected from 300 μm acute coronal mouse midbrain slices using a 1.0 mm^2^ tissue punch. Slices were visualized on an inverted fluorescence microscope during punching to confirm cell transduction *via* GFP reporter expression and to enrich for GFP-positive cells. RNA was extracted and reverse transcribed using RETROscript reverse transcription kit (Thermo Fisher Scientific). qPCR was performed and analyzed using the Applied Biosystems 7500 Real-Time PCR System Machine and software or using the Bio-Rad C1000 Touch Thermal Cycler with CFX96 Real-Time system and software, using Taqman gene expression assays for mouse DRD2 exon 2 to 3 (Mm00438541_m1), Vps35 (Mm00458167_m1), Rit2 (Mm0172749_mH), mGlu5 exon 7 to 8 (Mm01317985_m1), and GAPDH (Mm99999915_g1).

### *Ex vivo* slice biotinylation

Surface proteins in acute coronal slices were covalently appended with biotin as previously described by our laboratory (25, 29, 32, 89). Coronal slices were prepared from 5- to 9-week-old C57Bl/6J and *Pitx3*^*IRES-tTA*^*;TRE-HA-hM3Dq* mice (CNO and DHPG studies) or 4 to 6 weeks following viral injection (Drd2^fl/fl^, shVps35, shRit2, and mGlu5^fl/fl^ studies). All data were obtained from a minimum of three independent mice, from multiple striatal slices per mouse. Mice were sacrificed by cervical dislocation and rapid decapitation. Heads were immediately submerged in ice-cold *N*-methyl-d-glucamine cutting solution, pH 7.4 (20 mM Hepes, 2.5 mM KCl, 1.25 mM NaH_2_PO_4_, 30 mM NaHCO_3_, 25 mM glucose, 0.5 mM CaCl_2_·4H_2_O, 10 mM MgSO_4_·7H_2_O, 92 mM *N*-methyl-*D*-glucamine, 2 mM thiourea, 5M Na^+^ ascorbate, and 3 mM Na^+^ pyruvate). Brains were removed, glued to VT1200S Vibroslicer (Leica) stage, and submerged in ice-cold and oxygenated cutting solution. 300 μm coronal slices were prepared, and slices were hemisected along the midline prior to recovering in ACSF (125 mM NaCl, 2.5 mM KCl, 1.24 mM NaH_2_PO_4_, 26 mM NaHCO_3_, 11 mM glucose, 2.4 mM CaCl_2_·4H_2_O, 1.2 mM MgCl_2_·6H_2_O, pH 7.4) for 40 min at 31 °C. Hemislices were treated with the indicated drugs for the indicated times at 37 °C with constant oxygenation. Following drug incubations, slices were moved to ice, and surface DAT was labeled by biotinylation with the membrane-impermeant sulfo-NHS-SS-biotin as previously described (25, 29, 32, 33, 89). Striata were further subdissected to isolate VS and DS by cutting hemislices in a line from the lateral ventricle to lateral olfactory tract (as shown in Fig. 1*B*). Tissue was lysed in radioimmunoprecipitation assay buffer (10 mM Tris, pH 7.4; 150 mM NaCl; 1.0 mM EDTA; 0.1% SDS, 1% Triton X-100, 1% sodium deoxycholate) containing protease inhibitors (1.0 mM phenylmethylsulfonyl fluoride and 1.0 g/ml each leupeptin, aprotinin, and pepstatin) and phosphatase inhibitor cocktail V (EMD Millipore) (when evaluating protein phosphorylation). Tissue was disrupted by triturating sequentially through a 200 μl pipette tip, 22- and 26-gauge tech-tips and solubilized by rotating (30 min, 4 °C). Insoluble material was removed by centrifugation and the bicinchoninic acid protein assay (BCA, Thermo Fisher Scientific) was used to determine protein concentrations. Biotinylated proteins were quantitatively isolated with streptavidin agarose beads (Thermo) overnight with rotation (4 °C), at a ratio of 20 μg lysate to 30 μl streptavidin agarose beads, empirically determined to be in the linear range of bead saturation for biotinylated striatal proteins. Beads were washed with radioimmunoprecipitation assay buffer (three times), and recovered proteins were eluted in 2× Laemmli sample buffer supplemented with 100 mM DTT by rotation (30 min, room temperature). Both the eluted proteins and an aliquot of the original total lysate were resolved by SDS-PAGE, and proteins were detected and quantified by immunoblot as described in *Immunoblots and quantification*.

### Immunoblots and quantification

Proteins were resolved by SDS-PAGE, and proteins were detected and quantified by immunoblotting with the following antibodies.

#### DAT

Rat anti-DAT (MAB369; Millipore; 1:2000 dilution) or rabbit monoclonal anti-DAT (AB184451, Abcam, 1:1000 dilution for Fig. S2, *A* and *B* only), rabbit anti-TH (AB152, Millipore, 1:10,000 dilution), rabbit anti-pSer40 TH (AB5935, Millipore, 1:5000 dilution), mouse antitransferrin receptor (clone H68.4, Thermo Fisher; 13-6800). Secondary antibodies conjugated to horseradish peroxidase were all from Jackson ImmunoResearch, and immunoreactive bands were visualized by chemiluminescence using SuperSignal West Dura (Thermo Scientific). Nonsaturating immunoreactive bands were detected using either a VersaDoc 5000MP or a ChemiDoc imaging station (Bio-Rad) and were quantified using Quantity One software (Bio-Rad). Surface DAT was calculated by normalizing the biotinylated DAT signal to its corresponding amount of total DAT in that sample, detected in parallel from the same exposure of the same immunoblot. Raw surface DAT values are expressed as %total. For most experiments, DAT surface values following drug treatment were normalized to vehicle-treated samples, obtained from the contralateral hemislice in parallel. Surface DAT bands, and their corresponding total lysate, shown for each experiment were taken from the same exposure of the same immunoblot, and brightness/contrast levels were set identically for all blots. Boxed bands were cropped and rearranged for presentation purposes only.

### FSCV

Striatal hemislices were prepared as described for *ex vivo* slice biotinylation and recovered at 31 °C for a minimum of 1 h prior to recording in oxygenated ACSF supplemented with 500 μM sodium ascorbate. Glass pipettes containing a 7 μm carbon-fiber microelectrode were prepared and preconditioned in ACSF by applying triangular voltage ramps (−0.4 to +1.2 and back to −0.4 V at 400 V/s), delivered at 60 Hz for 1 h. Recordings were performed at 10 Hz. Electrodes were calibrated to a 1 μM DA standard prior to recording. Recording electrodes positioned in DS and DA transients were electrically evoked with a 250 μA, 0.2 ms, rectangular pulse delivered every 2 min with a concentric bipolar electrode placed ∼100 μm from the carbon fiber electrode. Data were collected with a three-electrode headstage, using an EPC10 amplifier (Heka) after low-pass filter at 10 kHz and digitized at 100 kHz, using Patchmaster software (Heka). A stable baseline was achieved after evoking six consecutive DA transients, after which experimental data were collected. Experiments ±L-741,626 were conducted on independent hemislices, and slices were superfused with ACSF ±L-741,626 for 15 min prior to initiating baseline recordings. Each biological replicate is the average of the three DA transients evoked following the initial six baseline transients., and a minimum of three independent mice were used to gather data from the indicated number of slices in each experiment. Data were analyzed in Igor Pro, using the Wavemetrics FSCV plugin (gift of Veronica Alvarez, National Institute on Alcohol Abuse and Alcoholism). Peak amplitudes were measured for each individual DA transient, and tau was calculated as 1/e according to the equation: y = y_0_ + A^((x-x^^_0_^^)/tau))^

### Mouse behavior

#### Locomotion

Mouse activity was individually assessed in photobeam activity chambers (San Diego Instruments) as previously described (32). Mice were placed in clean gromet-free cages and horizontal, vertical, and fine movement were measured in 5 min bins for 90 min total. In CE-158 studies, mice were tested over two consecutive days. Each session was comprised of 45 min habituation, I.P. injection, and 90 min recording. Mice were injected with vehicle (30% Kolliphor EL in sterile saline) on day 1 and the indicated CE-158 dose on day 2. CE-158 was prepared fresh on each day of experimentation.

#### Accelerating and fixed-speed rotarod

Mice were habituated to the behavior room in home cage for >30 min with ambient lighting and the RotaRod unit (UgoBasile 47,600) running at 4 RPM. Mice were weighed prior to testing

##### Accelerating rotarod

Mice were placed on the rod revolving at constant 4 RPM, and rod speed was then increased from 4 to 40 RPM over 5 min. Mouse latency to fall was measured over three consecutive trials and determined by either triggering the strike plate during a fall, or if the mouse made >1 consecutive passive rotation

##### Fixed speed

Mice were placed on the rod moving at the indicated speeds (20, 25, 30, 35, 40, and 45 RPM) and evaluated for two consecutive 60 s trials. Latency to fall was measured, or trial was stopped following >1 passive rotation.

#### Challenge/balance beam

One day prior to assay, mice were trained (five trials) to traverse a 1.0 m step-wise tapered (widths: 35, 25, 15, and 5 mm) elevated beam (catalog no.: 80306; Lafayette Neuroscience) at an incline of 15°. Training and assay were performed in a dark room with only one light source placed approximately 1.5 feet over the beam origin. A dark box with home-cage bedding was placed at the top of the incline. Mice were acclimated to testing room for >30 min with assay set up. On assay day, a challenge grid (custom 3D-printed; Thingiverse; catalog no.: 4869650) was placed over the beam, and mice traversed the beam in three independent trials. Traversals were video captured and scored for foot faults and traversal time, averaged over the first two completed trials or as paired analysis between first and second trial. Animal IDs were double blinded to both the experimenter and an independent scorer.

#### Grip strength

Mouse grip strength was measured using the Bioseb Grip Strength Test (BIO-GS3) equipped with mesh grip grid for mice. Mice were suspended by the tail over the mesh and allowed to grab the mesh with all four paws. The mouse was then pulled backward on the horizontal plane until it released from the mesh. The force applied, just before release, was recorded for three consecutive trials and averaged.

#### Gait analysis

Gait analysis assay was adapted from the study by Wertman *et al.* (90). Briefly, mouse fore paws and hind paws were dipped in orange and blue nontoxic tempera paint, respectively. The mice were placed in a 10 cm × 36 cm runway with 14 cm high foamboard walls and a dark box at the opposing end. Fresh and legal-size paper was placed on the bench top under the runway for each trial, and mice were placed on the paper at the open end of the runway and allowed to traverse to the closed box at the opposite end. Three trials were performed per mouse, and stride length, stride width, and toe spread were measured for both forelimbs and hindlimbs. Number of completed trials was also quantified. Mouse IDs were double blinded for both the assay and quantification.

### Statistical analysis

Data were analyzed using GraphPad Prism software (GraphPad Software, Inc). All data were assessed for normality, and nonparametric tests were applied in the event that data distribution was non-Gaussian. Outliers in a given dataset were identified using either Grubb’s or Rout’s outlier tests, with α or Q values set at 0.05 or 5%, respectively, and were removed from further analysis. Significant differences between two values were determined using either a one-tailed, two-tailed, or paired Student’s *t* test, as indicated. Differences amongst more than two conditions were determined using one-way or two-way ANOVA, as appropriate, and significant differences among individual values within the group were determined by post hoc multiple comparison tests, as described for each experiment. Power analyses were performed using G∗Power to determine sufficient sample sizes for behavioral assays given power (1 − β) = 0.9, α = 0.05, and effect sizes observed in previous and pilot studies. Minimum sample sizes were as follows: accelerating rotarod n = 5/group, fixed speed rotarod n = 5/group, total locomotion n = 6/group, grip strength n = 6/group, balance beam foot slips n = 5/group, balance beam traversal time n = 6/group, and gait analysis n = 5/group.

## Data availability

All data generated or analyzed during this study are included in this published article and its supporting information files.

## Supporting information

This article contains supporting information.

## Conflict of interest

The authors declare that they have no conflicts of interest with the contents of this article.
