# Supplementary material for: Presynaptic Gq-coupled receptors drive biphasic dopamine transporter trafficking that modulates dopamine clearance and motor function
Source: J Biol Chem. 2023 Jan 12;299(2):102900. doi: 10.1016/j.jbc.2023.102900 (PMC9943899; doi:10.1016/j.jbc.2023.102900)
Supplement: Supporting information [file mmc1.pdf]

Kearney et al

Supporting Information

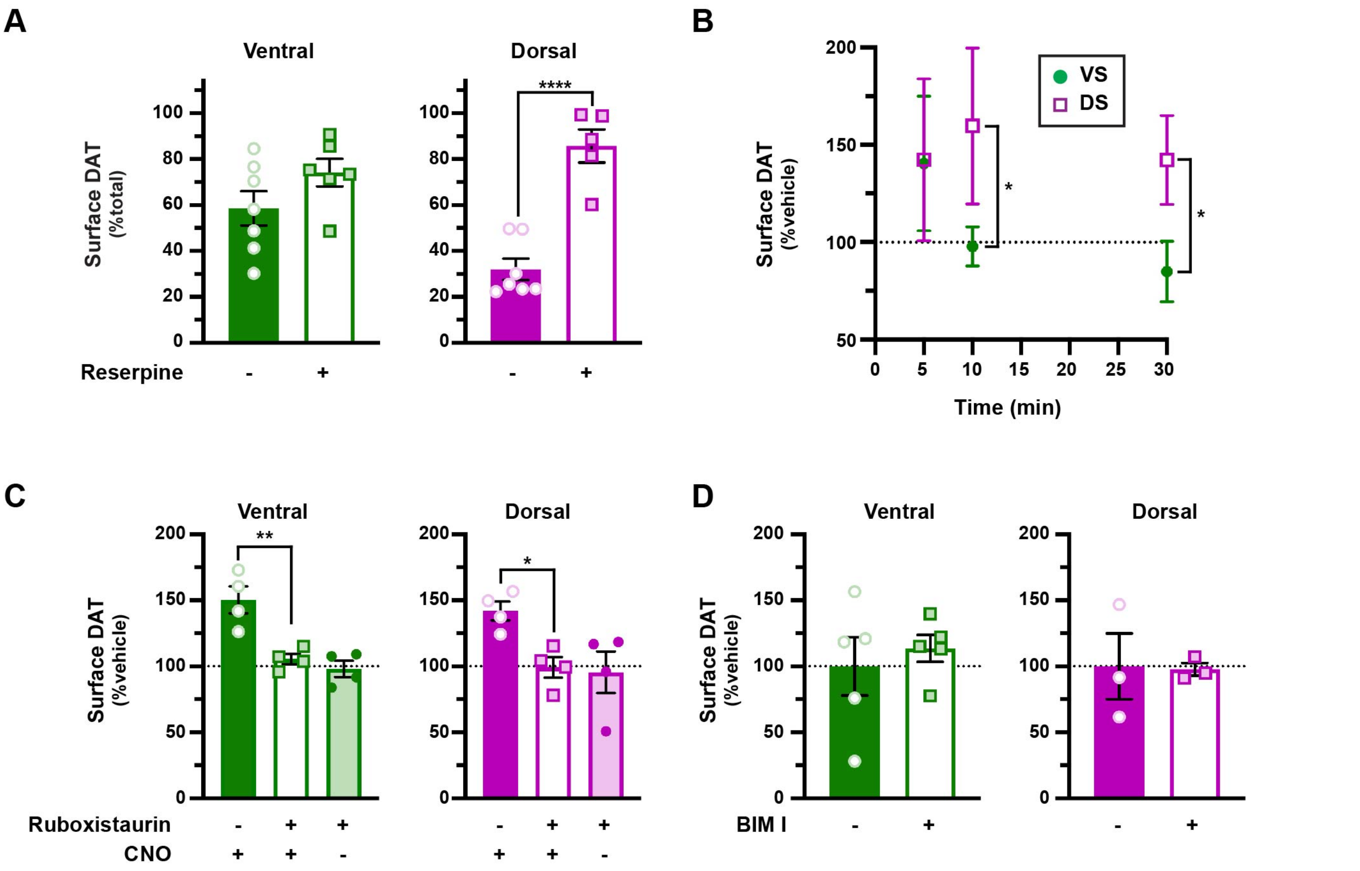

Figure S1. Mechanisms required for hM3Dq- and DRD2-stimulated DAT membrane delivery and retrieval. *Ex vivo striatal slice surface biotinylation.* Acute striatal slices were prepared from *Pitx3*$^{IRES\text{-}tTA}$*;TRE-hM3Dq* mice, were treated with the indicated drugs for the indicated times, and DAT surface levels were measured by slice biotinylation as described in *Experimental Procedures.* Dorsal and ventral striata were subdissected prior to solubilizing, as described in *Experimental Procedures.* Mean DAT surface levels are presented ±S.E.M. unless otherwise noted. **(A)** *Effect of reserpine treatment on basal DAT surface expression.* Mice were injected (I.P.) ±5.0 mg/kg reserpine 16 hrs prior to preparing slices, and 1.0μM reserpine or vehicle were included in the bath throughout the experiment. *Ventral:* p=0.14, two-tailed, unpaired Student's t test, n=7 (saline) and 6 (reserpine). *Dorsal:* ****p<0.0001, two-tailed, unpaired Student's t test, n=7 (saline) and 5 (reserpine). **(B)** *DRD2 activation increases DAT surface expression:* Slices were treated ±170nM sumanirole for the indicated times. Average DAT surface levels are presented as %vehicle-treated levels ±S.D. Two-way ANOVA: Interaction: $F_{(2, 23)}$ =2.92, p = 0.07; Time: $F_{(2, 23)}$ =2.08, p=0.15, Region: $F_{(1, 23)}$ =12.55, **p=0.002. DAT surface expression remained significantly more elevated in dorsal striatum than ventral striatum following 10 min(*p=0.02) and 30 min (p=0.02) treatment times, Bonferroni's multiple comparisons test, n=4-5 (ventral) and 5 (dorsal). **(C)** *hM3Dq-stimulated DAT insertion requires PKCβ activity.* Slices were pretreated ±ruboxistaurin (50nM, 30 min, 37°C) and hM3Dq was stimulated ±CNO (500nM, 5 min). *Ventral:* One-way ANOVA: $F_{(2,9)}$ =15.10, **p=0.001; Ruboxistaurin significantly blocked CNO-stimulated DAT insertion, **p=0.004, Bonferonni's multiple comparison test, n=4. *Dorsal:* One-way ANOVA: $F_{(2,9)}$ =5.54, *p=0.03; Ruboxistaurin significantly blocked CNO-stimulated DAT insertion, *p=0.04, Bonferonni's multiple comparison test, n=4. **(D)** *Effect of BIM I on DAT surface expression.* BIM I treatment alone had no significant effect on DAT surface levels in either ventral (p=0.55) or dorsal (p=0.93) striata (two-tailed, unpaired Student's t test, n=5 (ventral) and n=3 (dorsal).

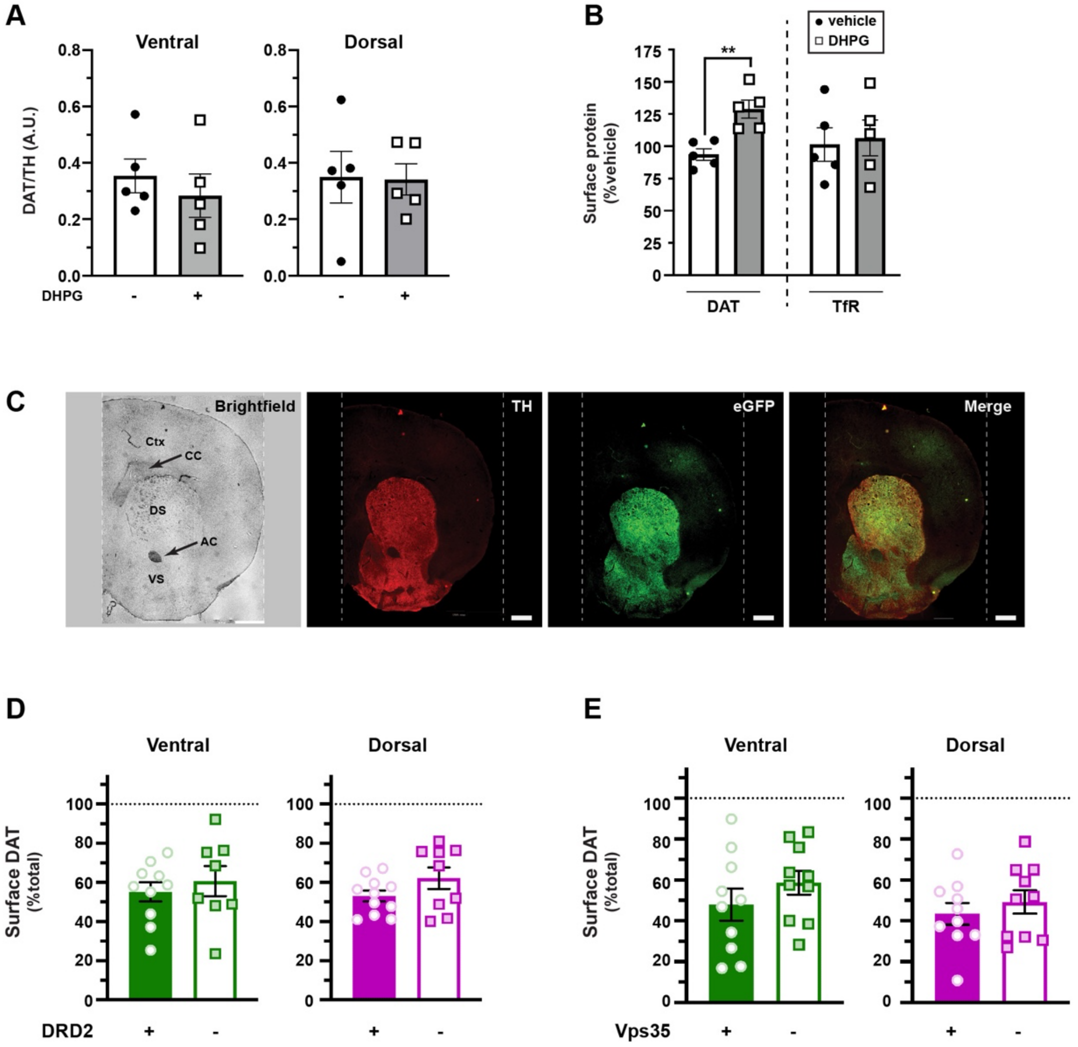

Figure S2. A. Effect of DHPG treatment on total DAT expression. **A.** *Quantitative immunoblotting.* Striatal slices were treated ±1μM DHPG, 5', 37°C and total DAT in dorsal (*left)* and ventral (*right)* striata was quantified, normalized to TH levels to account for DAergic terminal variability among slices. Values are average DAT/TH ±S.E.M. Acute DHPG treatment had no effect on total DAT in either dorsal (p=0.94) or ventral (p=0.50) striata, two-tailed, unpaired Student's t test, n=5. **B. Effect of DHPG treatment on general endocytic trafficking.** *Ex vivo striatal slice surface biotinylation*. Acute striatal slices were prepared from wildtype mice, treated ±1μM DHPG, 5 min, 37°C, and both DAT and TfR surface levels in the ventral striatum were measured in parallel samples by slice biotinylation as described in *Experimental Procedures.* DHPG significantly increased surface DAT (p=0.003**)** but did not significantly affect TfR surface levels (p=0.79), two-tailed, unpaired Student's t test, n=5. **C.** *AAV9 viral spread validation*. *Pitx3$^{IRES\text{-}tTA}$* mouse VTA were bilaterally injected with AAV9-TRE-shVPS35-eGFP. Brains were harvested and sectioned (25μm) 4 weeks post-injection, and were stained for TH (red) and eGFP (green) as described in *Experimental Procedures*. Images were captured at Bregma +1.54mm in brightfield, red, and green channels as indicated. A representative hemisphere is shown. Note robust eGFP expression in both dorsal and ventral striatum that co-localizes with TH. Ctx (cortex), DS (dorsal striatum), VS (ventral striatum), AC (anterior commissure), CC (corpus collosum). Scale bar = 500μm. Dashed line indicates edge of acquired image. **D, E. Effect of conditional DRD2 and Vps35 silencing on basal DAT surface expression.** *Ex vivo striatal slice surface biotinylation*. Acute striatal slices were prepared mice injected with the indicated viruses, and DAT surface levels were measured by slice biotinylation as described in *Experimental Procedures*. Mean DAT surface levels in ventral (left) and dorsal (right) striata are presented as %total DAT ±S.E.M. **D.** DRD2auto silencing: *Pitx3$^{IRES\text{-}tTA}$;DRD2$^{fl/fl}$* mouse VTA were bilaterally injected with AAV9-TRE-Cre. $DRD2_{auto}$ silencing had no significant effect on basal DAT surface levels in either ventral (p=0.54) or dorsal (p=0.13) striata, as compared to Cre-injected, *Pitx3$^{IRES\text{-}tTA}$* controls, two-tailed, unpaired Student's t test, n=8-10 (ventral) and n=9-11 (dorsal). **E.** Vps35 silencing. *Pitx3$^{IRES\text{-}tTA}$* mouse VTA were bilaterally injected with either AAV9-TRE-eGFP or AAV9-TRE-shVps35. Conditional Vps35 silencing had no significant effect on basal DAT surface levels in either ventral (p=0.28) or dorsal (p=0.46) striata, as compared to control-injected mice (two-tailed, unpaired Student's t test, n=10 (ventral) and n=10 (dorsal).

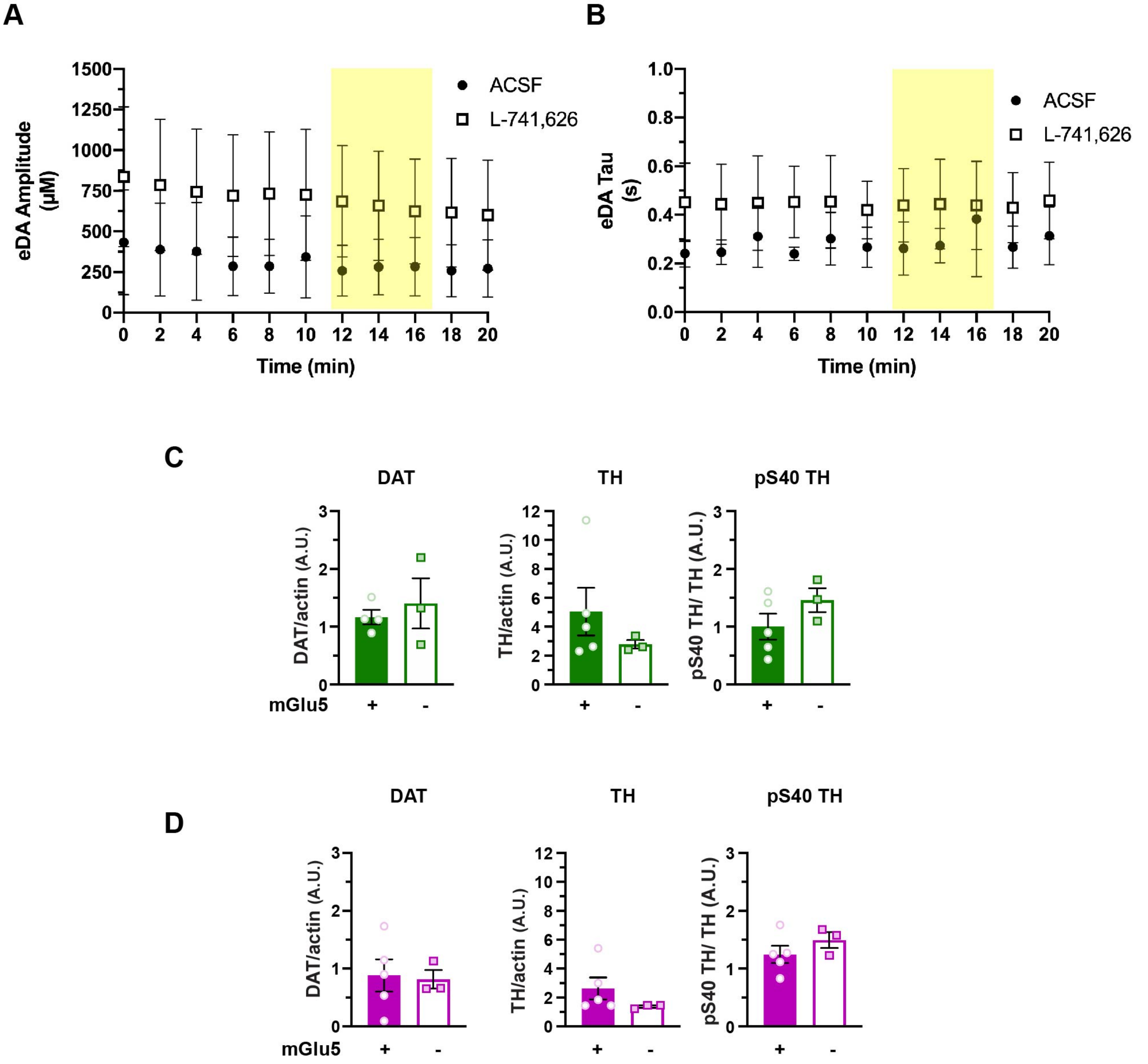

Figure S3. A,B. Effect of time and DRD2 antagonist on DA transient amplitude and tau in dorsal striatum. *Ex vivo FSCV.* Striatal hemi-slices were prepared as described in *Experimental Procedures*. A single hemi-slice was transferred to the recording chamber and perfused with either ASCF or L-741,626, 15 min, 37°C, prior to evoking DA release. DA release was electrically evoked every 2 min, as described in *Experimental Procedures.* Six DA transients were evoked to establish baseline, and data from transients 7-9 were averaged for each hemi-slice (yellow shaded). *Left: DA transient amplitude (µM).* L-741,626 pre-treatment, significantly increased DA amplitudes as compared to ACSF pretreatment, and there was no significant change in amplitude over time. Two-way ANOVA, time: p=0.66, drug: p<0.0001, interaction: p>0.999, n=9. *Right: Decay tau (sec).* L-741,626 pre-treatment, significantly increased DA clearance (decreased tau) compared to ACSF pretreatment, and there was no significant change in amplitude over time. Two-way ANOVA, time: p=0.92, drug: p<0.0001, interaction: p=0.94, n=9. **C,D. Effect of conditional mGlu5 silencing on total DAT, TH, and pS40TH.** *Quantitative immunoblotting. $Pitx3^{IRES\text{-}tTA}$;$mGlu5^{fl/fl}$* mouse VTA were bilaterally injected with either AAV9-TRE-eGFP or AAV9-TRE-Cre, and DAT, TH, and pS40-TH levels were measured in lysates from ventral and dorsal striata. Mean protein levels were normalized to either actin loading controls (for DAT and TH) or pan-TH (for pSer40-TH), are presented as average ±S.E.M. **(C)** *Ventral striatum:* Conditional mGlu5 silencing had no significant effect on either total DAT (p=0.57, n=4 [eGFP) and 3 [Cre]), total TH (p=0.38, n=5 [eGFP) and 3 [Cre]), or pSer40-TH (p=0.22, n=5 [eGFP) and 3 [Cre]), two-tailed, unpaired, Student t test. **(D)** *Dorsal striatum:* Conditional mGlu5 silencing had no significant effect on either total DAT (p=0.87), total TH (p=0.35), or pSer40-TH (p=0.31), two-tailed, unpaired, Student's t test, n=5 (eGFP) and 3 (Cre).

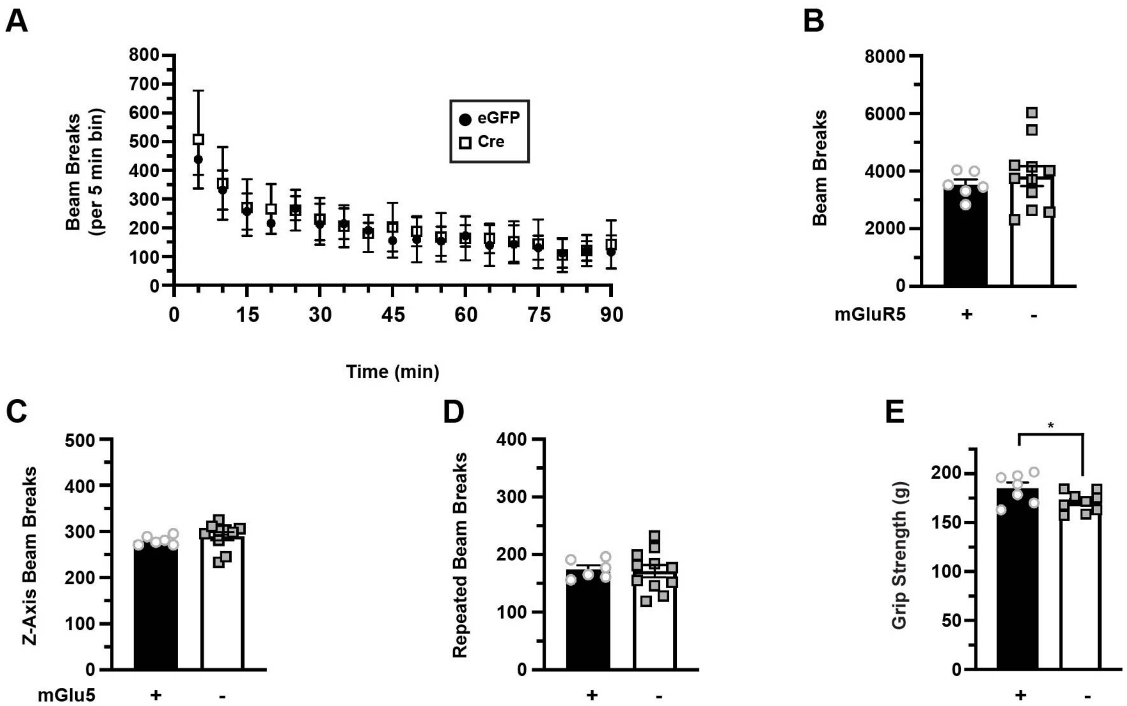

Figure S4. Effect of conditional mGlu5 silencing on mouse baseline locomotion and grip strength. *Mouse locomotor studies. Pitx3$^{IRES\text{-}tTA}$;mGlu5$^{fl/fl}$* mouse VTA were bilaterally injected with either AAV9-TRE-eGFP or AAV9-TRE-Cre and baseline locomotor activity was monitored in photobeam activity chambers as described in *Experimental Procedures.* **(a)** *Total horizontal locomotion over time*. **(b-d)** *Averaged data.* Average total movement measured throughout the recording session, ±S.E.M. Conditional mGlu5 silencing had no significant effect on horizontal locomotion (p=0.55, **b**), vertical movement (p=0.95, **c**), or fine movement (p=0.85, **d**), two-tailed, unpaired, Student t test, n=6 (eGFP) and n=11 (Cre). **(e)** *Grip Strength:* Mouse grip strength was assessed as described in *Experimental Procedures.* Average data are presented as force required (g) to drive mouse release ±S.E.M. Conditional mGlu5 silencing significantly decreased mouse grip strength (p=0.04), two-tailed, unpaired, Student t test, n=6 (eGFP) and n=11 (Cre).

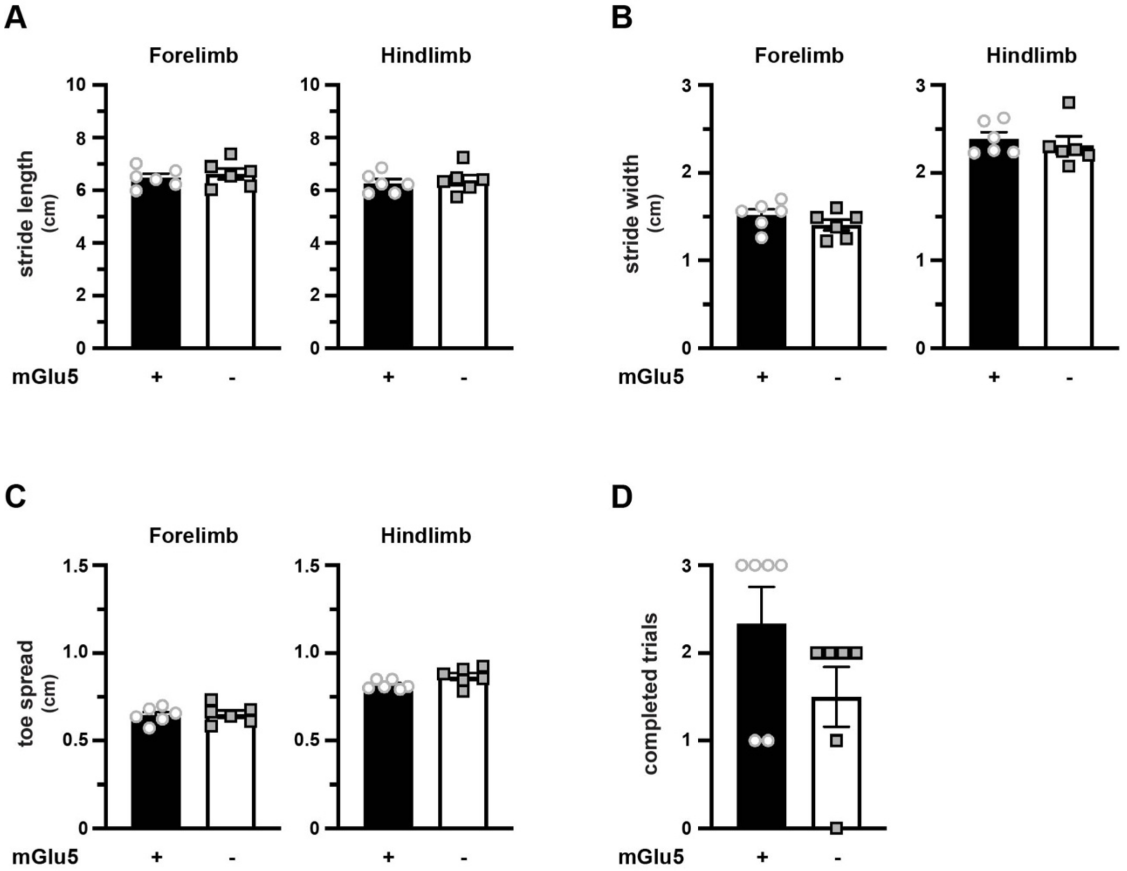

Figure S5. Effect of conditional mGlu5 silencing on mouse gait. *Pitx3$^{IRES\text{-}tTA}$;mGlu5$^{fl/fl}$* mouse VTA were bilaterally injected with either AAV9-TRE-eGFP or AAV9-TRE-Cre and mouse gait was assessed as described in *Experimental Procedures.* Conditional mGlu5 silencing had no significant effect on either **(A)** stride length in the forelimb (p=0.60) or hindlimb (p=0.66), **(B)** stride width in the forelimb (p=0.21) or hindlimb (p=0.56), **(C)** toe spread in the forelimb (p=0.83) or hindlimb (p=0.06), or **(D)** total completed trials (p=0.16) (two-tailed, unpaired, Student t test, n=6 (eGFP) and n=11 (Cre).

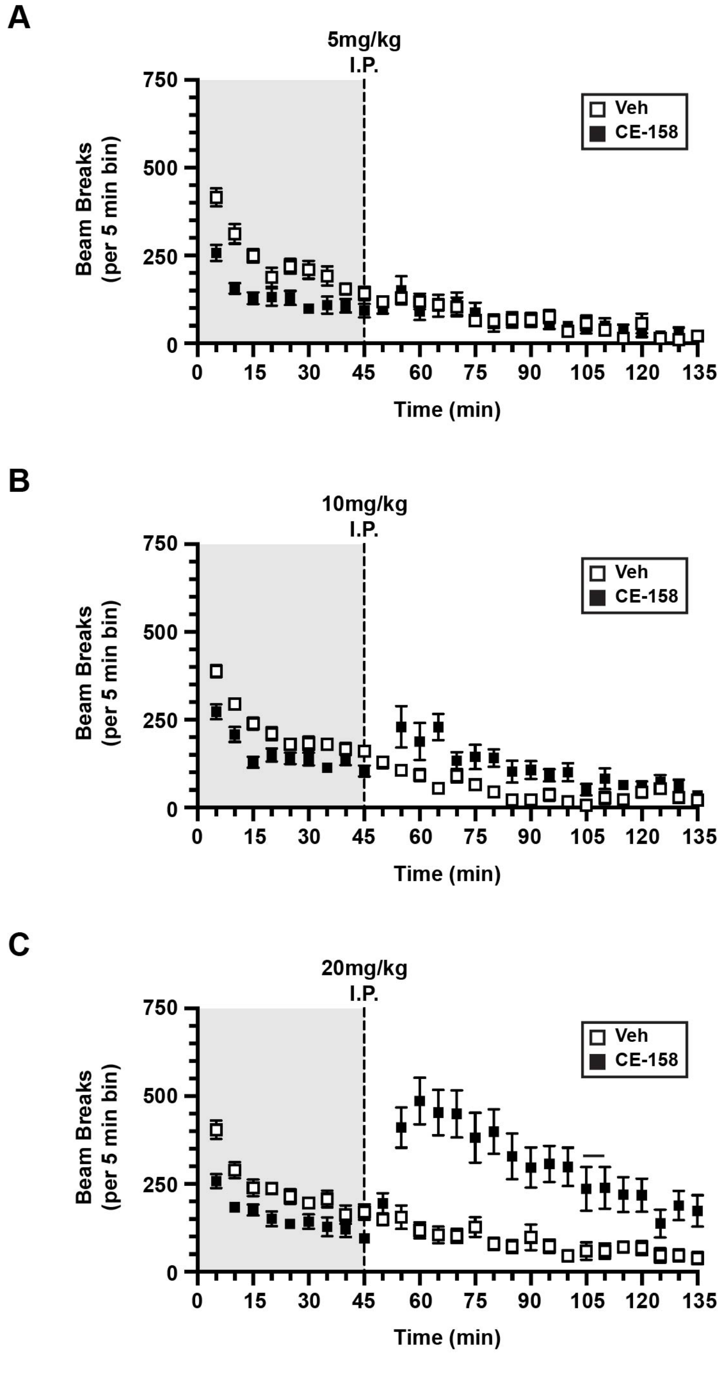

Figure S6. Effect of DAT inhibitor CE-158 on wildtype mouse horizontal locomotion. *Mouse locomotor studies. WT* mouse horizontal locomotor activity was monitored in photobeam activity chambers as described in *Experimental Procedures.* Mice were habituated to the test cage for 45 min before I.P. injection ±CE-158. Horizontal locomotion was measured over the subsequent 90min. Vehicle and CE-158 data were collected from the same animals over 2 consecutive days of testing. **(A)** *5mg/kg* no significant effect of drug. Two-way Repeated Measures ANOVA: Interaction: $F_{(26, 468)}$ =5.322, ****p < 0.0001; Time: $F_{(26, 468)}$ =39.66, ****p < 00001; Drug: $F_{(1, 18)}$ =3.208, p=0.0901; Subject: $F_{(18, 468)}$ = 15.59, ****p < 0.001; n=10 **(B)** *10mg/kg* no significant effect of drug. Two-way Repeated Measures ANOVA: Interaction: $F_{(26, 468)}$ =9.648, ****p < 0.0001; Time: $F_{(26, 468)}$ =36.02, ****p < 00001; Drug: $F_{(1, 18)}$ =1.057, p=0.3176; Subject: $F_{(18, 468)}$ = 16.74, ****p < 0.001; n=10. **(C)** *20mg/kg* significantly increases horizontal locomotion. Two-way Repeated Measures ANOVA: Interaction: $F_{(26, 468)}$ =15.98, ****p < 0.0001; Time: $F_{(26, 468)}$ =9.570, ****p < 00001; Drug: $F_{(1, 18)}$ =3.208, **p=0.0026.18; Subject: $F_{(18, 468)}$ = 18.74, ****p < 0.001; n=10.
